# Social reference managers and their users: A survey of demographics and ideologies

**DOI:** 10.1371/journal.pone.0198033

**Published:** 2018-07-11

**Authors:** Pei-Ying Chen, Erica Hayes, Vincent Larivière, Cassidy R. Sugimoto

**Affiliations:** 1 Department of Information and Library Science, School of Informatics, Computing, and Engineering, Indiana University, Bloomington, Indiana, United States of America; 2 Carolina State University Libraries, Raleigh, North Carolina, United States of America; 3 École de bibliothéconomie et des sciences de l’information, Université de Montréal, Montréal, Québec, Canada; 4 Observatoire des sciences et des technologies (OST), Centre interuniversitaire de recherche sur la science et la technologie (CIRST), Université du Québec à Montréal, Montréal, Québec, Canada; 5 Department of Informatics, School of Informatics, Computing, and Engineering, Indiana University, Bloomington, Indiana, United States of America; KU Leuven, BELGIUM

## Abstract

Altmetric indicators are increasingly present in the research landscape. Among this ecosystem of heterogeneous indicators, social reference managers have been proposed as indicators of broader use of scholarly work. However, little work has been done to understand the data underlying this indicator. The present work uses a large-scale survey to study the users of two prominent social reference managers—Mendeley and Zotero. The survey examines demographic characteristics, usage of the platforms, as well as attitudes towards key issues in scholarly communication, such as open access, peer review, privacy, and the reward system of science. Results show strong differences between platforms: Mendeley users are younger and more gender-balanced; Zotero users are more engaged in social media and more likely to come from the social sciences and humanities. Zotero users are more likely to use the platform’s search functions and to organize their libraries, while Mendeley users are more likely to take advantage of some of the discovery and networking features—such as browsing papers and groups and connecting with other users. We discuss the implications of using metrics derived from these platforms as impact indicators.

## Introduction and background

The scholarly communication system has been transformed over the past two decades by the digital age, Web 2.0 applications, and open scholarship initiatives. In recent years, social media platforms have significantly increased the online visibility of scholarly products: with more than 4.5 million researchers on ResearchGate and 11 million users on Academia.edu, there is considerable evidence of the increased usage of social media platforms for the creation, evaluation, and dissemination of scholarship [1]. In parallel, demands by research funders and administrators for indicators of societal impact [2–4] have generated wide interest among scholars in evaluating how social media data may be used as a means for measuring scholarly impact beyond the academy [5].

Citations have historically served as the dominant measurement for the impact of scholarly publications [6]. Citation counts, however, serve as indicators of papers’ impact on other scholars, and are not indicative of their impact on the broader public [7]. New sets of metrics, grouped under the umbrella of “altmetrics”, propose new indicators of scholarly impact based on interactions on social media platforms [8]. Introduced in 2010 by Jason Priem and his colleagues, “altmetrics” claims to “expand our view of what impact looks like” [8] by identifying online communities and tracking their online engagement with scholarly content [8,9]. Often considered as a subset of webometrics due to its reliance on open application program interface (API) usage data, altmetrics has sought to move beyond the traditional citation measurement of journal article by tracking online social media activities [10,11]. These activities include the appraisal of scholarly articles through the act of mentioning them online; using these articles to create new scholarly objects; or accessing, curating, and saving scholarly objects online [9]. There is wide variety in the tools that are used to perform each of these actions; however, one of the most commonly used online tools to access, save, and curate documents are social reference managers.

Social reference managers were created to assist researchers in the referencing process when writing research articles and formatting citations—functions that “traditional” reference managers such as Endnote performed and to which “social” functions were added. Social reference managers allow users to save, bookmark, download, and share research articles with other scholars that may be of interest or use to them. The two most popular social reference managers are Zotero and Mendeley. Both platforms provide users with tools to leave comments, rate papers, cite entries, share work, create subject tags, and join built-in social networking communities among scholars of varying disciplines [5]. Referred to as academic social networking services [12], these social reference management systems provide a means for scholars to interact and share academic documents with other researchers.

Data from these applications can also be used to reveal readership behavior and create indicators of documents’ online use. Mendeley is particularly useful in this regard as it provides anonymized usage data for reportedly 2.5 million registered users via an open API [13]. Founded in August 2008, Mendeley began as a free software, but was purchased by Elsevier for $76 million in April 2013 [1]. Although negative responses from the research community followed Elsevier’s purchase of Mendeley among open access advocates, Mendeley has argued that its mission to facilitate data sharing and make scientific research more openly available has remained much the same since its conception [14]. Mendeley currently provides a desktop and web platform with 2GB of free web storage space for the management, saving, bookmarking, and sharing of research papers between scholars on the web. The main features include automatic extraction of metadata, smart-filtering, tagging, annotating, and full-text search. Mendeley also offers various bibliographic file formats for exporting scholarly materials, including EndNote XML, BibTex, and RIS [15]. In addition to reference management, Mendeley also features discovery of new references via “Literature Search” or “Mendeley Suggest” and research network via creating or joining groups [16].

Zotero was released in 2006 by the Center for History and New Media at George Mason University, which continues to maintain it as an open-source software platform. Zotero provides 300 MB of free cloud storage for users to store and save research articles. Available since the latest release as a standalone desktop application for users of all browsers [17], Zotero automatically extracts and saves bibliographic references when viewing a book, article, or other scholarly object via browser extensions [18]. Offering similar features to Mendeley, Zotero provides users with automatic extraction of metadata and full-text search, which can be organized by creating collections or assigning tags [18]. Users may also join private or public closed/open membership groups to collaborate, discuss, and share research online [19]. Scholarly materials may be exported in common bibliographic data formats, including BibTex, BibLateX, RefWorks, and RIS [20]. Since Zotero is open source, users contribute new ideas and improvements to the software on an ongoing basis. The forums on the Zotero website are frequently updated regarding the most recent changes or improvements to the software with the latest 5.0 version being released in July 2017 [21].

The size of the user base for Zotero was estimated at around 620,000 in 2011 [22]. However, interpretation of this figure is complicated by the fact that Zotero never required users to register their accounts before downloading the software, making it impossible to know whether each download is a unique or duplicate user [22]. Furthermore, Zotero does not provide an API to download data and estimate user accounts from the existing metadata. As a result of these limitations, no large-scale study of Zotero users has been conducted.

Mendeley API provides users’ disciplines and academic status/occupations for each article. Using these data, previous research investigated the disciplinary and occupational distributions of Mendeley users and their patterns of information behavior. It showed that the majority of Mendeley users are doctoral students, followed by master’s students and postdocs [23,24]. Correlations between Mendeley readership counts and citations for the same articles varies by occupation: correlations are highest when citations are compared with Mendeley reader counts from research-oriented occupations and lowest when limited to those of librarians and students [24]. These variations suggest that Mendeley readers reveal important indicators of use by professionals and students that are not captured by citations. This is reinforced by user surveys, which demonstrated that Mendeley users primarily use the platform to support professional and educational activities [25]. The heterogeneity of users in terms of disciplinary and occupational composition explains the discrepancies observed when examining the relationship between Mendeley reader counts and other impact indicators [26].

Mendeley provides full access for document-based analysis, which has led to several coverage studies, i.e., what proportion of sets of documents are indexed in the database. These studies have shown that the coverage of articles in Mendeley is generally higher than in other social platforms, such as CiteULike [27,28] and Twitter [29], albeit with some variations by research field and publication venue. For example, rates of coverage tend to be lowest in the humanities [30] and highest in biomedicine [31], with certain subfields, such as bibliometrics [32], having high coverage, as well as specific venues, including *Public of Library Science* (PLoS) [33], *Science*, *Nature* [28], and *Journal of the American Society for Information Science and Technology* (JASIST) [34]. The moderate positive correlations (.45-.50) between Mendeley readership counts and citation counts across disciplines [24,28,30,33,34] suggest that Mendeley readership counts reflect related but non-identical indicators of scholarly impact. Until recently, no API allowed for similar coverage analyses of Zotero.

Despite the increasing use of readership counts as indicators of papers’ usage—and their integration into aggregated altmetric indicators, such as the Altmetric Attention Score calculated by the company Altmetric, little is known on the processes and individuals that generate these traces of readership. In order to meaningfully interpret such indicators, we must better understand the motivations underlying the interactions as well as the characteristics of those making the interactions. Generalizations about the value of articles are increasingly made on the basis of these counts. Yet, without a proper understanding of the characteristics of the population underlying the metrics, it is difficult to understand on whom the impact was made. To address this, we conducted a survey of Zotero and Mendeley users to understand not only their demographic characteristics and how they utilize the platforms, but also on their attitudes towards key issues in scholarly communication, such as open access, peer review, privacy, and the reward system of science. The results of this study inform our understanding of these platforms and the degree to which interactions on these platforms can be used to measure broader impacts.

## Methods

This study has been approved by the Institutional Review Board at Indiana University. (IRB Study #1512031107).

### Survey design

Two online surveys were designed specifically for Mendeley and Zotero users, each composed of 22 question stems in three parts, including demographic information, user behavior, and meaning and motivation, with a study and consent information sheet provided in the beginning. Socio-demographic information collected included gender, age, current place of residence, level of education, occupation/status, and affiliated disciplines. Questions regarding user behavior ranged from the length of use; pattern of use over time; platform primarily used; group membership; to frequency of performing different kinds of tasks including discoverability, adding and organizing, re-using, and networking. Meaning and motivation questions included rating the importance of social reference managers in fulfilling different tasks/activities to level of engagement in reading, citation, curation, and sociability, as well as their ideological positions toward scholarly communication. Answers to the questions regarding level of importance were marked on a Likert scale from 1 (not at all important) to 5 (extremely important). Likewise, answers regarding level of agreement are marked from 1 (strongly disagree) to 5 (strongly agree).

For the purpose of comparison, the wording and the order of the questions for the two surveys were held constant except when the names of the respective social reference managers and terminology exclusively used by each were mentioned, e.g., platforms (Mendeley: online/desktop vs Zotero: standalone/for Firefox) as well as the unit of readership counts (Mendeley readership vs Zotero library). Moreover, as Zotero was released earlier than Mendeley, Zotero survey has an extra option of “more than seven years” in the question about the length of use.

### Survey distribution

The surveys were hosted on Qualtrics and distributed from mid-April to early June 2016. Due to different modes of cooperation and levels of concern over privacy, the two surveys were distributed in different ways. A representative sample of more than 26,000 user emails were provided by Mendeley to send invitations and reminders with the unique survey link embedded, as well as to trace the response rate. On the other hand, the anonymous link to the Zotero survey was disseminated by Zotero through various channels including library-related listservs (between April 14–18), the Zotero blog (April 13), Twitter (April 18, 22, 29 and May 6, 13, 17), LinkedIn, Trellis, as well as the Open Scholarship Initiative 2016 Conference (April 19–22). In total, 1214 responses from Mendeley users and 796 from Zotero users were received, of which 767 and 529 (respectively) were complete cases. The response rate for the Mendeley survey is 4.61%. No response rate could be calculated for Zotero.

While the low response rate and the use of convenient sample may raise some methodological concern, several studies have pointed out that low response rates are not necessarily correlated with nonresponse error [35]. A comparison of Mendeley user profiles on occupation and discipline with previous survey studies [12,25] and studies based on Mendeley readership statistics gathered through API [23,36] showed that the respondents in our study are well aligned with the disciplinary mapping of users in the Mendeley directory released in 2013, with an exception of an underrepresentation of basic science. As to occupation, despite some variations in the proportion of each category due to research designs and methodological limitations, previous studies generally agreed, in consistence with ours, that Mendeley is predominantly used by student, researcher, and professor (see S1 Table). The use of convenience sample for Zotero users provides an initial, exploratory perspective on Zotero users.

### Survey analysis

The two separate raw datasets were downloaded from Qualtrics in comma-separated values (CSV) file format, then read and combined in RStudio, with a new variable “platform” to record the platforms from which the responses were collected. In addition to performing Chi-square tests of independence on all the nominal/ordinal questions, logistic regression was also applied to questions regarding motivation to use, level of engagement, and ideological positions to further examine the effects of other socio-demographic variables. To do this calculation, 5-point Likert scales were converted into a binary variable by assigning 1 to the original value 4’s (agree/important) and 5’s (strongly agree/extremely important) and 0 to the value 1’s (strongly disagree/extremely unimportant) and 2’s (disagree/unimportant) while dropping the value 3’s (neither agree/important nor disagree/unimportant). This is a limitation of the study, as 3 is a legitimate response and dropping these responses lowered the amount of responses which could be analyzed. However, the decision was made to render the results more comparable by highlighting stances closer to both ends of the spectrum. The gender variable was recoded into “male,” “female,” and “other” with the original “other” and “prefer not to answer” categories being combined into “other” due to the limited cases in the two categories. The full model includes platform, level of education, affiliated discipline, occupation, gender, and age as controlled variables to test the respective explanatory power.

## Results

### Socio-demographic characteristics of participants

Mendeley reported roughly equal rates in terms of gender; whereas Zotero was much more male-skewed (66% male) (Table 1). The gender difference between the two platforms is statistically significant (X^2^ = 44.37, p < .001). Both the majority of Mendeley and Zotero users are in their mid- to late-thirties, with an average age of 36.28 years for the former and 38.31 years for the latter, and the difference in age is statistically significant (t = -3.43, p < .001). One plausible explanation for the age gap is that Zotero was released two years earlier than Mendeley, which also suggests that users are aging with the platform.

**Table 1:** Socio-demographics of survey respondents.

	Mendeley		Zotero		Total	
*Gender*						
Male	378	(49.3%)	349	(66.0%)	727	(56.1%)
Female	378	(49.3%)	164	(31.0%)	542	(41.8%)
Prefer not to answer	9	(1.2%)	13	(2.5%)	22	(1.7%)
Other	2	(0.3%)	3	(0.6%)	5	(0.4%)
*Age* ^*a*^						
Mean	36.28	(10.55)	38.31	(10.45)	37.11	(10.55)
Median	34		36		35	
Min	17		18		17	
Max	85		69		85	
*Occupation*						
Student	266	(34.7%)	125	(23.6%)	391	(30.2%)
Researcher	191	(24.9%)	103	(19.5%)	294	(22.7%)
Professor	160	(20.9%)	109	(20.6%)	269	(20.8%)
Practitioner	74	(9.6%)	134	(25.3%)	208	(16.0%)
Lecturer	46	(6.0%)	25	(4.7%)	71	(5.5%)
None of the above	30	(3.9%)	33	(6.2%)	63	(4.9%)
*Level of education*						
Master	346	(45.1%)	267	(50.5%)	613	(47.3%)
Doctorate	322	(42.0%)	222	(42.0%)	544	(42.0%)
Bachelor	87	(11.3%)	32	(6.0%)	119	(9.2%)
High school	12	(1.6%)	8	(1.5%)	20	(1.5%)
*Affiliated discipline*						
Social Sciences	162	(21.1%)	117	(22.1%)	279	(21.5%)
Life Sciences	207	(27.0%)	70	(13.2%)	277	(21.4%)
Arts & Humanities	62	(8.1%)	143	(27.0%)	205	(15.8%)
Others	72	(9.4%)	85	(16.1%)	157	(12.1%)
Engineering	84	(11.0%)	26	(4.9%)	110	(8.5%)
Physical Sciences	46	(6.0%)	21	(4.0%)	67	(5.2%)
Psychology	52	(6.8%)	7	(1.3%)	59	(4.6%)
Computer Sciences	34	(4.4%)	22	(4.2%)	56	(4.3%)
Environmental Sciences	34	(4.4%)	13	(2.5%)	47	(3.6%)
None	6	(0.8%)	20	(3.8%)	26	(2.0%)
Mathematical Sciences	8	(1.0%)	5	(0.9%)	13	(1.0%)

Notes

^a^ The numbers in parentheses are standard deviation.

About 90% of the respondents hold a master’s degree or above, indicating that they are more educated than the general public, with Zotero users being marginally more well-educated (X^2^ = 11.46, p < .01). In line with their advanced education level is the occupation or status to which they belong. Students, researchers, and professors are overall the three largest user groups across two surveys, but the composition of occupations between Mendeley and Zotero is significantly different (X^2^ = 69.14, p < .001). In particular, more than 25% of Zotero users are practitioners, defined as those work in professional fields such as librarianship, nursing, or medicine that typically require a professional degree or an advanced certificate, in contrast with less than 10% of their Mendeley counterparts. There is also a significant difference regarding users’ affiliated disciplines (X^2^ = 164.35, p < .001). While the life sciences, social sciences, and engineering are the three disciplines that Mendeley users are mostly affiliated with, Zotero users are mostly affiliated with arts and humanities, followed by social sciences and life sciences. Also noted is that 16% of Zotero users reported to be affiliated with “other” disciplines, of which 70% were shown to be in library and information science after manual inspection.

### User behavior

More than 60% of Mendeley users have been using the product for 1–4 year, and 90% less than four years. In contrast, Zotero respondents have, on average, a longer history of using the product, with nearly 55% using it for more than four years (S2 Table). Although the differences could be largely attributed to the longer history of Zotero in the market, it is worth noting that 21% of Zotero users have been using it for almost the same length of time since the release of Zotero, indicating either their loyalty to the product, or their early adoption of new technologies. The patterns of use over time are significantly different between Mendeley and Zotero users (X^2^ = 38.67, p < .001) (S3 Table). While 70% of Zotero users report either a constantly increased or relatively constant use over time, 40% of Mendeley users report that their use fluctuates.

Although cooperating with others is one of the features distinguishing Mendeley and Zotero from other reference managers, nearly half of the Mendeley users and more than one third of Zotero users join neither public nor private groups (S4 Table). The difference is statistically significant regarding the group membership between Mendeley and Zotero users (X^2^ = 35.673, p < .001) despite the fact that both are more likely to join private than public groups. Also noted is that 21% of Zotero users are part of both private and public groups, compared to 11% of their Mendeley counterparts.

**Fig 1:**
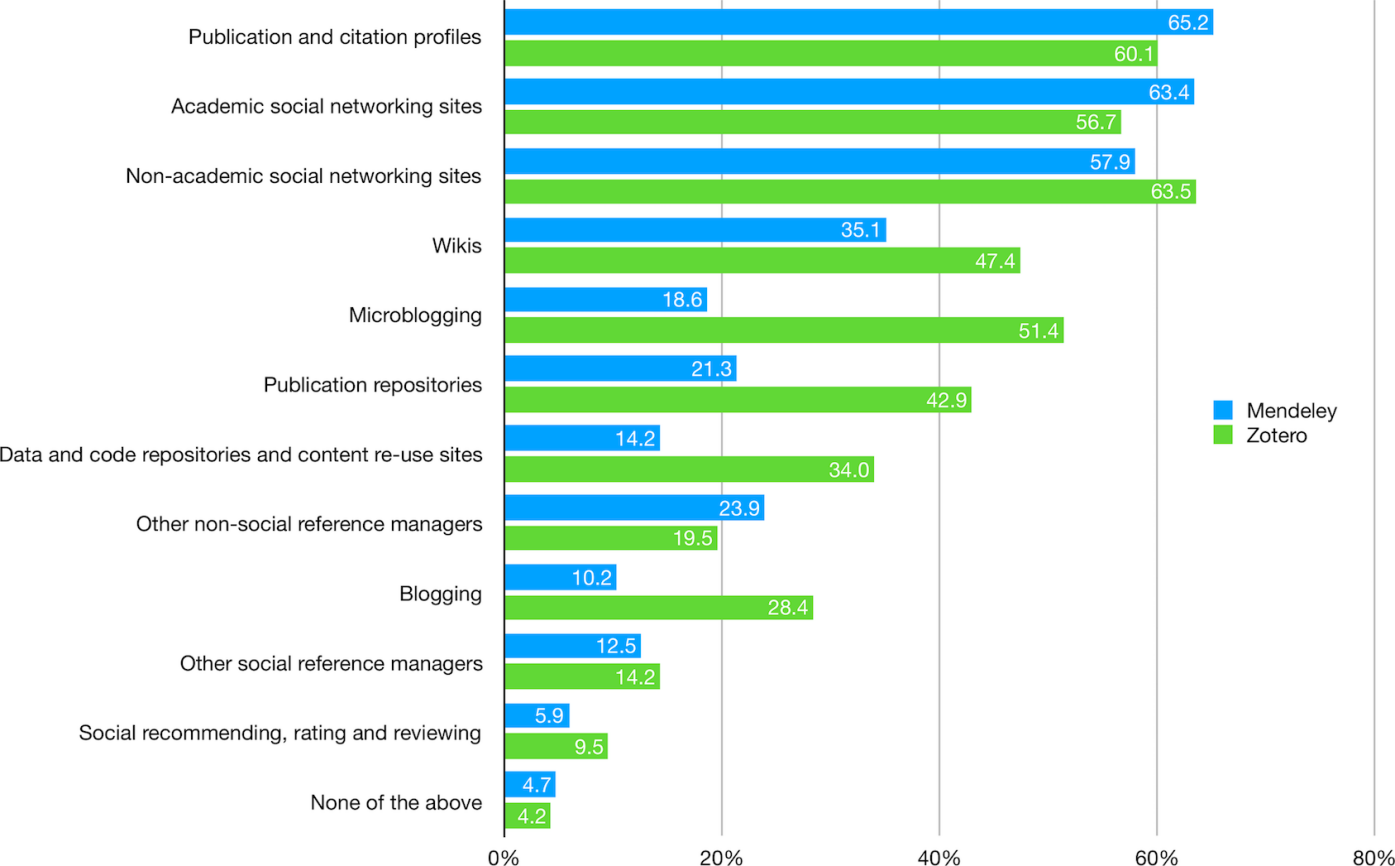
Tools for professional purposes (i.e., those associated with your vocation rather than for personal use).

In addition to Mendeley and Zotero, respondents reported the use of diverse tools for professional purposes. Publication and citation profiles (e.g., Google Scholar, ORCID), non-academic (e.g., Facebook, LinkedIn) and academic social networking sites (e.g., ResearchGate, Academia.edu), and to some extent, Wikis (e.g., Wikipedia), have been used extensively by both Mendeley and Zotero users (Fig 1; S5 Table). However, the two groups differ in the use of publication (e.g., arXiv, SSRN, institutional repository) as well as data and code repositories (e.g., GitHub, Figshare, SlideShare), with the percentages of reported use among Zotero users twice as much as those of Mendeley users. Moreover, Zotero users are much more actively engaged in blogging and microblogging activities. Finally, compared with tools mentioned above, social recommending, rating, and reviewing (e.g., F1000Prime, Reddit) are the least utilized by both groups, probably indicating their relatively short history as a new form of scholarly communication.

**Fig 2:**
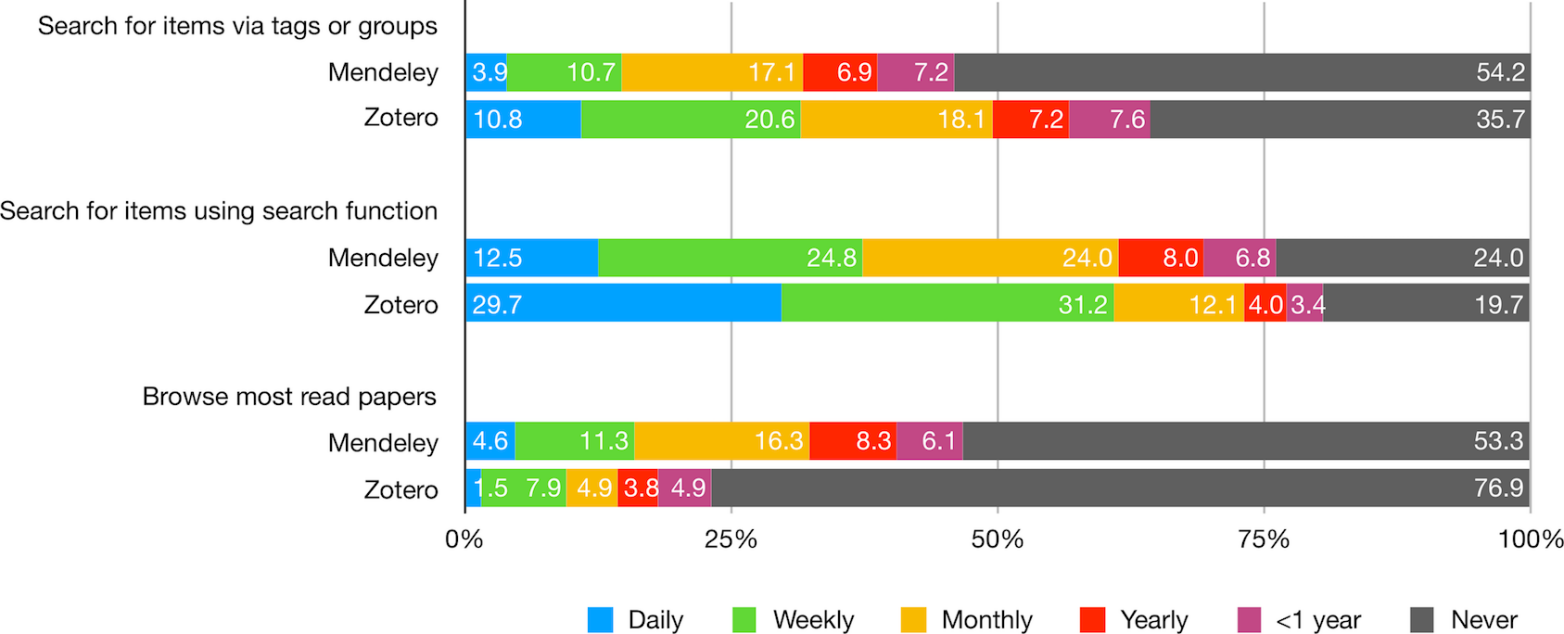
How often do you do the following discoverability tasks on Mendeley/Zotero?

Overall, Zotero users performed discoverability tasks more often than Mendeley users, with more than 60% of them, compared to less than 40% of Mendeley users, using the search function at least weekly. Other discoverability features, however, were much less utilized: 54% of Mendeley and 36% of Zotero users reported that they never search via tags or groups; moreover, 53% of Mendeley and 77% of Zotero users reported that they never browse the most read papers (Fig 2; S6 Table).

**Fig 3:**
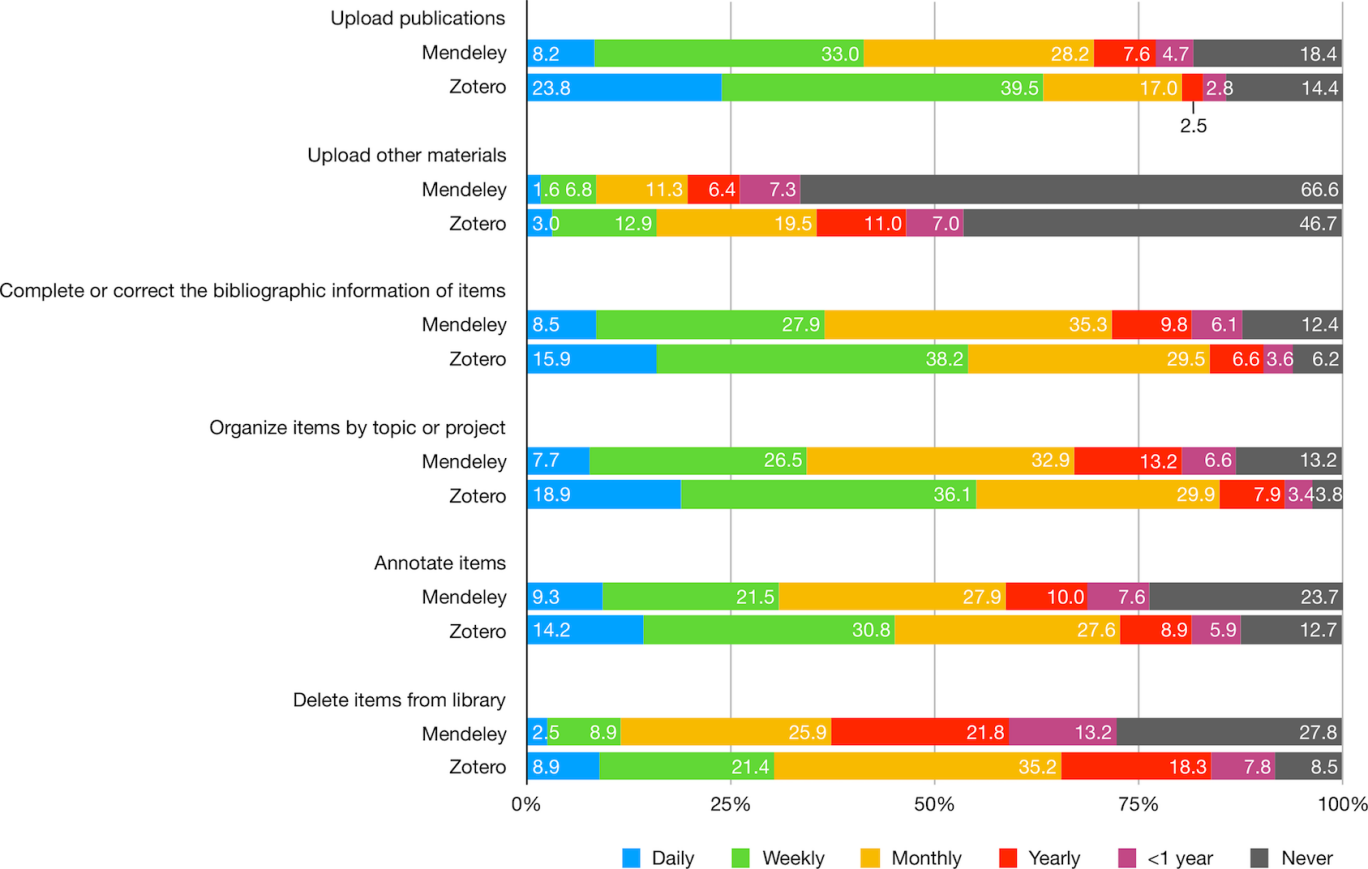
How often do you do the following adding and organizing tasks on Mendeley/Zotero?

Both groups performed adding and organizing tasks quite frequently, but this was more prominent for Zotero users. Almost two third of Zotero users and 40% of Mendeley users uploaded publications at least weekly. However, nearly 70% of Mendeley and 50% of Zotero users never upload other materials such as datasets, figures, or videos. Other organizational tasks, such as correcting bibliographic information or annotating were done on a monthly basis by the majority of Mendeley and Zotero users. The two user groups differed mainly in the frequency in which they deleted items from their libraries: Zotero users were much more frequently to do so than Mendeley users (Fig 3; S7 Table).

**Fig 4:**
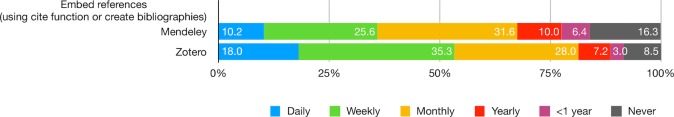
How often do you do the following re-using task on Mendeley?

The re-using task was frequently performed by both Mendeley and Zotero users, with two third of Mendeley and more than 80% of Zotero users doing this on a monthly or more frequent basis. Zotero users seem to be more dedicated in using social reference managers to embed references, as more than half of them did such task at least weekly, compared to less than 40% of Mendeley users. This is in line with the more professional occupation base of Zotero (Fig 4; S8 Table).

**Fig 5:**
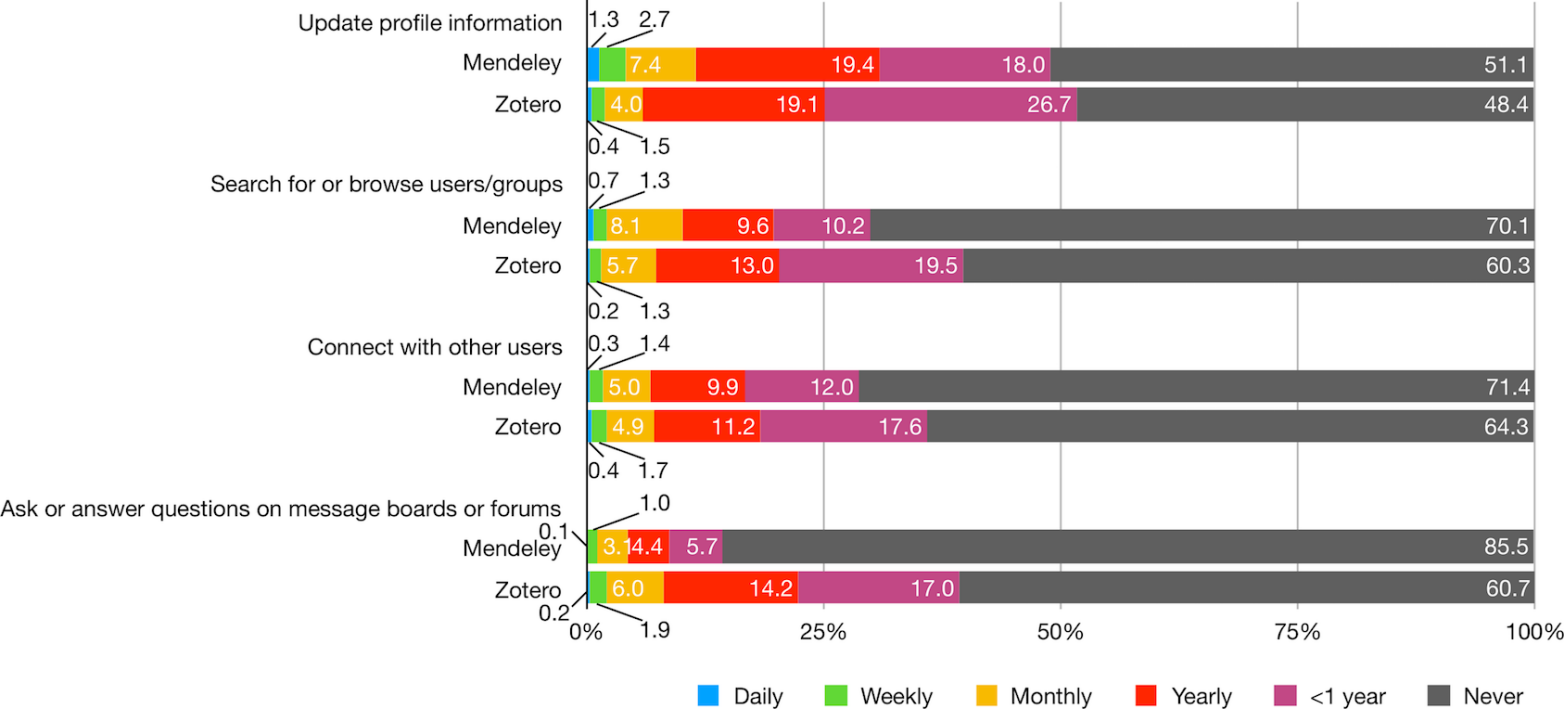
How often do you do the following networking tasks on Mendeley/Zotero?

Compared with the other three functionalities, networking tasks were seldom performed by both groups, with 50–80% of Mendeley users and 50–65% of Zotero users never performed tasks such as updating profile information, searching for or browsing users/groups, connecting with others, and asking or answering questions on message boards or forums (Fig 5; S9 Table). Overall, Zotero users engage in networking activities relatively more frequently than Mendeley users except for updating profile information.

### Motivation to use

**Fig 6:**
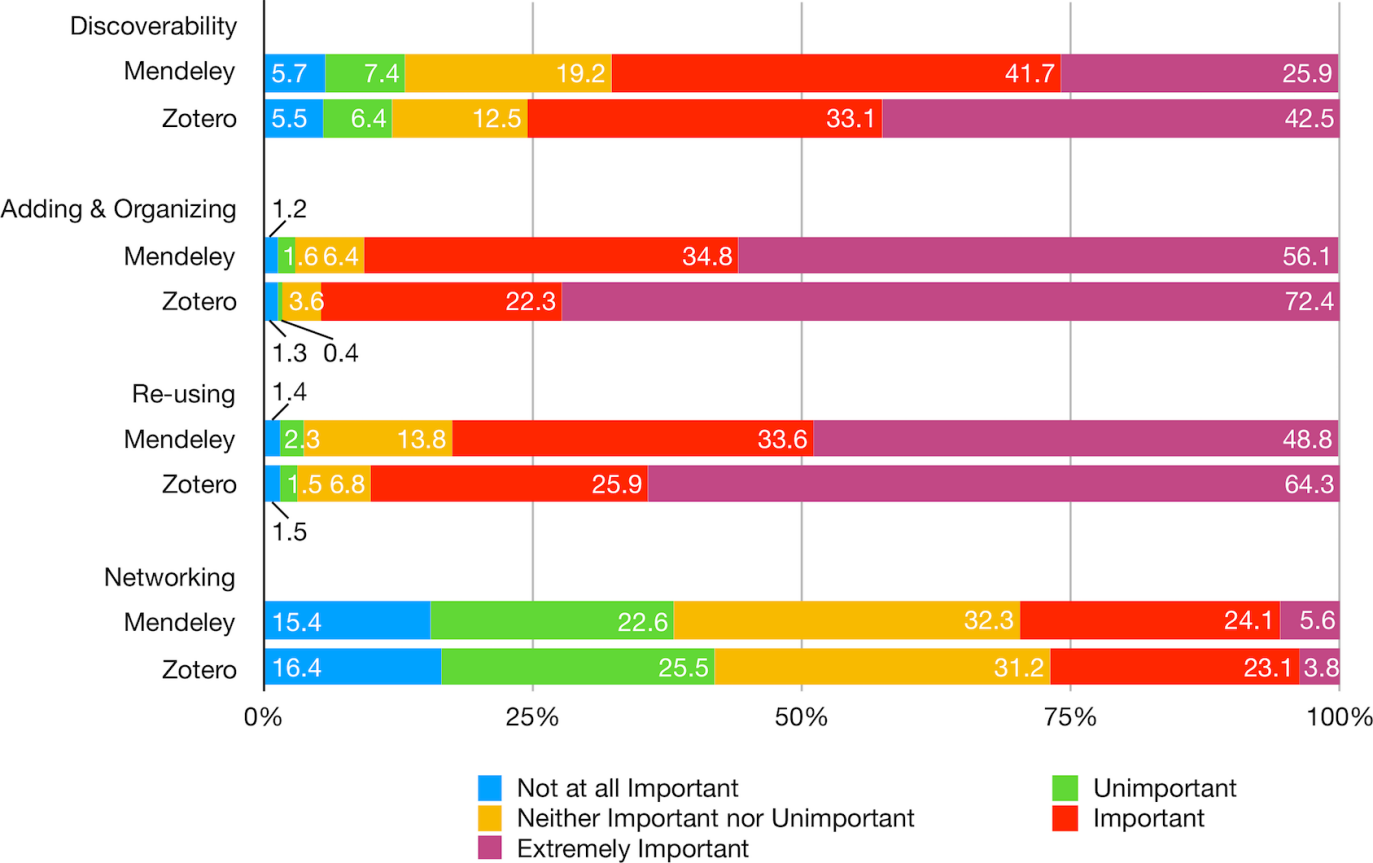
Please rate the importance of the following types of features.

Overall, features of discoverability, adding and organizing, and re-using are deemed important across two user groups in spite of statistically significant differences (p < .001) with Zotero users being more likely to stress these features as extremely important (Fig 6; S10 Table). Nonetheless, the networking feature seems negligible for both user groups with no statistically significant differences found (X^2^ = 3.80, p = 0.43): 40% of all the respondents rate this as unimportant to not at all important and 32% as neither important nor unimportant. While respondents holding a bachelor’s degree are more likely to consider discoverability important than those with a master’s degree and professors consider adding and organizing features important than students, the overall effects of platform, education level, affiliated discipline, occupation, gender, and age are not statistically significant across these features except for networking, where education and occupation do hold certain explanatory power (p < .001) (S11 Table). On the one hand, professors, lecturers, and practitioners are more likely than students to consider networking an important feature. On the other hand, compared to respondents with a master’s degree, high school graduates are more likely to consider networking important whereas those with a doctorate degree are less likely to think so.

**Fig 7:**
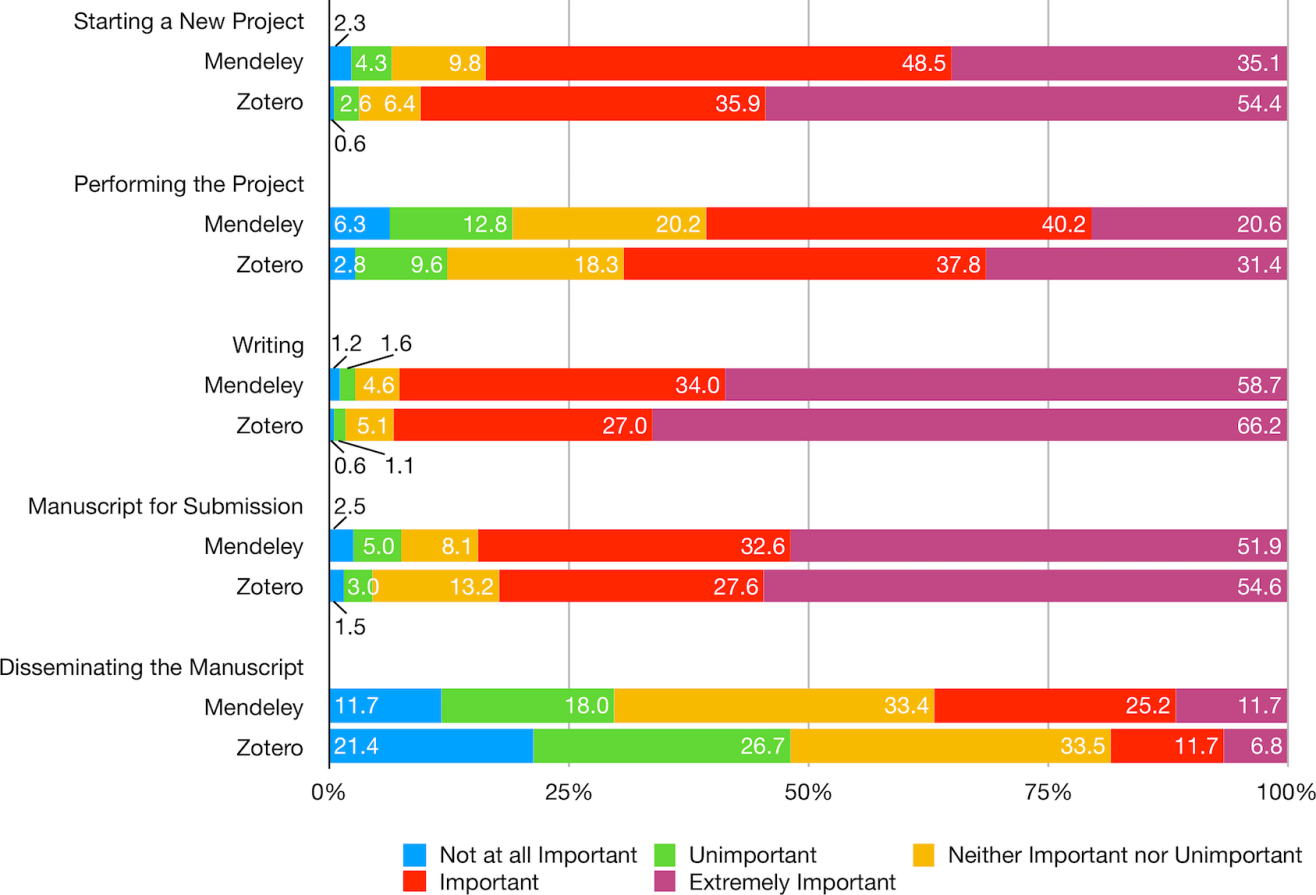
How important is Mendeley/Zotero to you at various stages of a research project?

Respondents generally consider social reference managers important at various stages of a research project from starting a research or assignment, collecting and analyzing data, to writing and preparing the manuscript for submission (Fig 7; S12 Table). The only exception is at the stage of post-publication, i.e., disseminating the manuscript: 30% of Mendeley users and nearly half of Zotero users consider the role of social reference manager either unimportant or not at all important. Overall, Zotero users are more likely than Mendeley users in rating the social reference manager as important in initiating and performing the project as well as preparing the manuscript for submission but less likely at the post-publication stage (p < .001) (S13 Table).

Other than platform, the overall effects of education, affiliated discipline as well as occupation are also found to be statistically significant at the stage of performing the project and disseminating the manuscript (S13 Table). For example, social reference managers are rated as less important at both stages for respondents with a doctorate degree than with a master’s degree. Compared to respondents affiliated with social sciences, those affiliated with arts and humanities and with engineering are more likely to consider social reference managers indispensable in performing the project, whereas those affiliated with psychology are less likely to think so. Respondents affiliated with engineering and environmental sciences are also more likely to recognize their significance in post-publication. Moreover, they are more likely to be considered important by researchers, practitioners, and those with other occupations than students in collecting and analyzing data and by lecturers in disseminating their manuscript.

**Fig 8:**
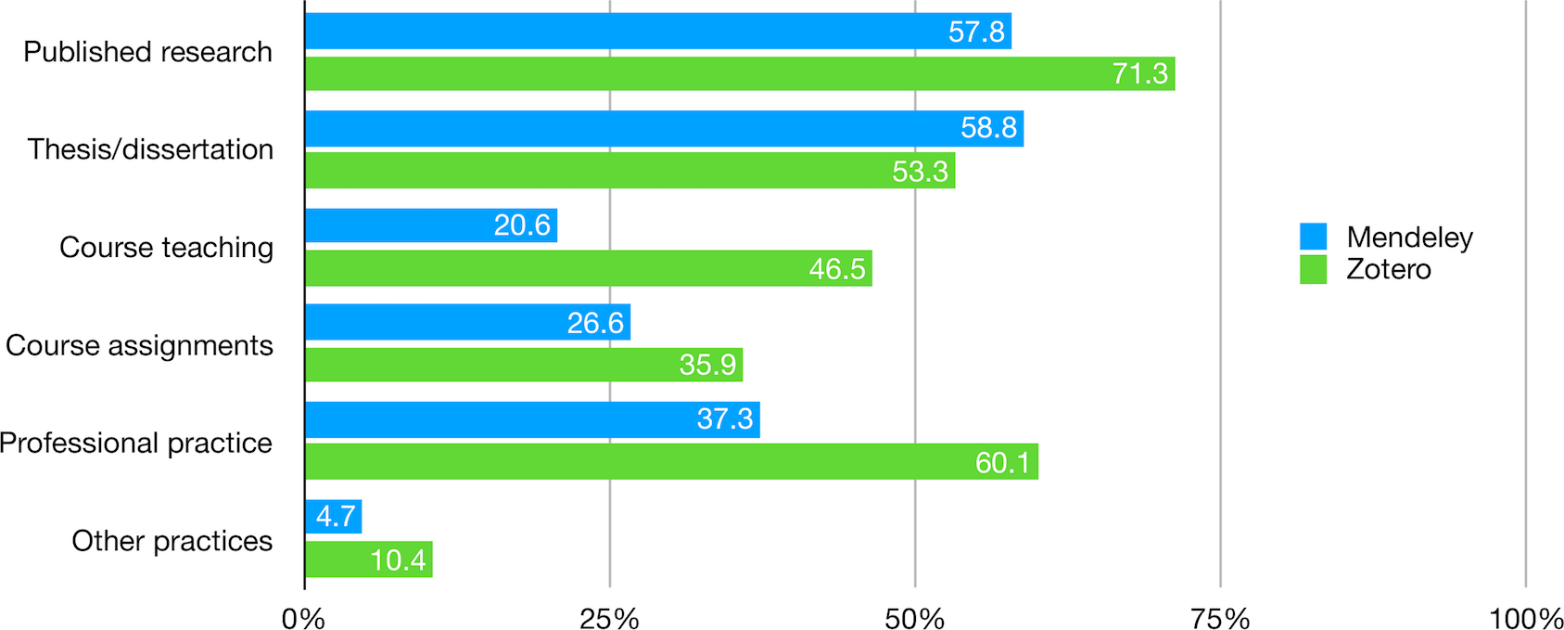
My Mendeley/Zotero library has contributed to my: (select all that apply).

In addition to research-related activities, Mendeley and Zotero users also reported various activities the social reference managers contribute to, including thesis/dissertation, courses teaching and assignments, as well as professional practice. Overall, Mendeley is used mainly for published research and thesis/dissertation whereas the use of Zotero is more evenly distributed across activities with higher share of users (Fig 8; S14 Table). In particular, Zotero is more likely than Mendeley to contribute to published research, course teaching, course assignments, as well as professional and other practices. The difference between the two groups is not statistically significant in writing thesis or dissertation (S15 Table).

Other than platform, the overall effects of level of education, affiliated discipline, occupation, and age are significant on the activities of Mendeley and Zotero users (S15 Table). Compared to those with a master’s degree, respondents with a doctorate degree are more likely to use social reference managers for their published research but less likely for course assignments, whereas respondents with a bachelor’s degree are in the reverse position. Activities regarding published research, theses/dissertations, and professional practice also differ by respondents’ affiliated disciplines, most notably computer sciences, life sciences, physical sciences. Compared to those in social sciences, respondents in the aforementioned disciplines are more likely to use Mendeley/Zotero for published research but less likely for theses/dissertations. Also worth noting is that social reference managers are more likely to contribute to professional activities for respondents affiliated with arts and humanities, life sciences, and other disciplines but less likely with computer sciences. Not surprisingly, non-students are more likely than students to use them for published research (professor, lecturer, researcher), course teaching (professor, lecturer, practitioner), and professional practice, but less likely for theses/dissertations and course assignment (researcher). Lastly, as people get older, they are more likely to use social reference managers for course teaching and professional practice but less likely in theses/dissertations and course assignments.

### Level of engagement

**Fig 9:**
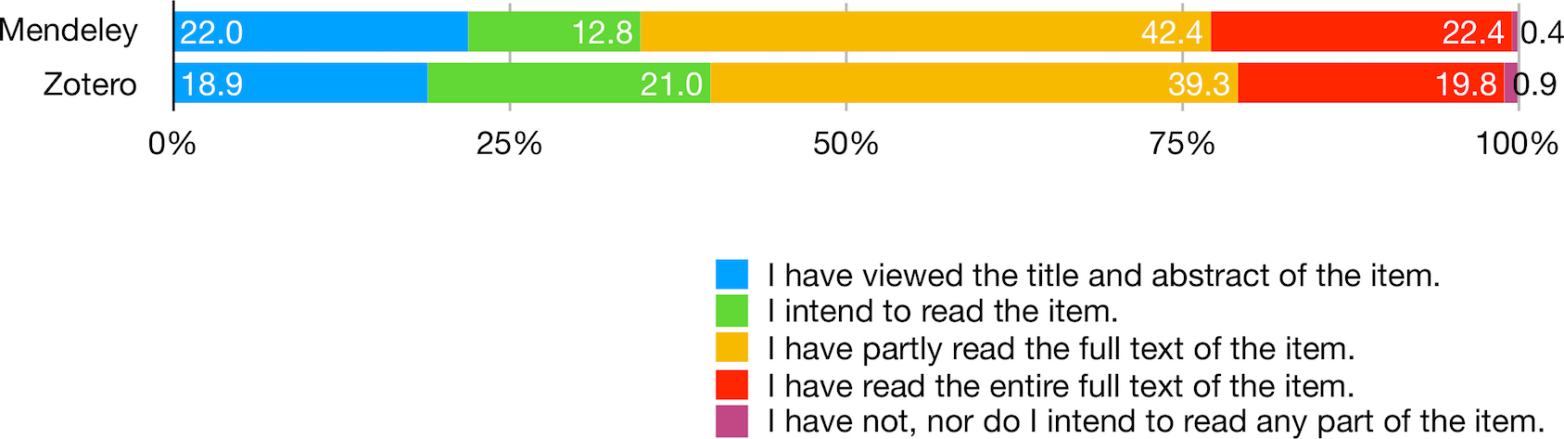
Consider the last item you remember having added to your library. Which of these statements best characterizes your level of engagement with it?

**Fig 10:**
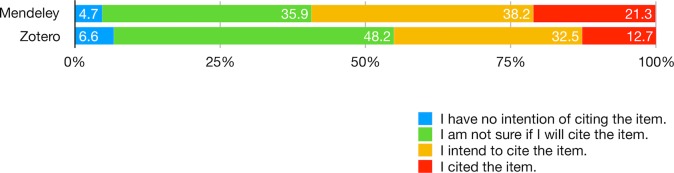
Consider the last item you remember having added to your library. Which of these statements best characterize your citation behavior?

Mendeley and Zotero users differ in their level of engagement with social reference managers in terms of reading, citation, curating, and social behaviors. In reflecting upon the last item added to the library, the differences are statistically significant in terms of reading (X^2^ = 17.79, d.f. = 4, p < .01) and citation (X^2^ = 29.62, d.f. = 3, p < .001). Not only did Mendeley users report higher rates of having viewed the title and abstract of the item and having read the full text either partly or entirely (Fig 9; S16 Table), they also report higher rates of both intending to cite and have cited the item (Fig 10; S17 Table). In contrast, more than 20% of Zotero users reported only the intent to read and nearly half were not sure if they will cite the item. Overall, a fifth of users (from both platforms) reported having read the item in full (identical to the proportion of users who only viewed the title and abstract of the item). This suggests that adding an item precedes reading. The patterns are consistent with their general reading and citation behaviors. 60% and 56% of Mendeley users respectively reported having read and cited most of the items in their library, whereas only 51% and 30% of Zotero users claimed the same (S18 Table).

Selection of items was fairly similar across platforms. Self-saving appears to be common across platforms, particularly in Zotero, where more than half of respondents indicated that they add all of their own publications to their library. Nearly 30% of Mendeley users neither agreed nor disagreed with this statement, possibly because they do not have publications to add to the library, leading to the ambiguity of the response. The overall quality of the items was not a deciding factor for either group—either in adding or deleting items from the library. Of the two, Mendeley users were slightly more concerned with the quality of items that were added, while over half of Zotero users disagreed that they only add items of high quality. As implied by the earlier questions, the social aspects of social reference managers are relatively unimportant, particularly for Zotero users. Users were unconcerned about saving to the library as a signaling device to the community and were not concerned about constructing a library for the good of the community. They also tended not to be interested in networking capabilities. The main value-add for them was uni-directional: allowing them to keep current with research rather than researchers in the field (S18 Table).

Compared with Mendeley users, Zotero users are not only less likely to have read and cited most of the items in their libraries but also have lower intention to cite them as well (S19 Table). Although they curate their libraries more often, quality is less of a concern for them in adding and deleting items (S20 Table). They also engage with Zotero at a more individual level: adding items is neither a signal to other scholars nor for the good of the community. Likewise, they are less likely to consider Zotero a networking tool to keep in contact with other scholars or to keep current on research (S21 Table).

The overall effect of the level of education is not statistically significant on users’ level of engagement except for adding items as signals to other scholars, where respondents holding a doctorate degree are less likely than those with a master’s degree to do so. By contrast, occupation is a strong predictor of users’ level of engagement in reading, citation, and social behaviors. Compared to students, non-students are less likely to engage in reading (lecturer, researcher, practitioner) and citation (researcher, practitioner) and have lower intention to cite as well (professor, researcher, practitioner, none). Nonetheless, they are more likely to take sociability into account, such as considering adding items either a signal to other scholars (lecturer) or for community good (lecturer, practitioner) and acknowledging social reference managers a tool for networking with other researchers (professor, lecturer, practitioner).

The overall effect of discipline is significant in reading, intent of citing, as well as self-saving to the library. Compared to respondents in social sciences, people in life sciences and physical sciences are more likely to have read most of the items in their library, whereas those affiliated with arts and humanities, engineering, life sciences, other or no discipline are less likely to intend to cite the items in their library. In addition, people in life sciences are more likely to add all of their publications to the library—thus taking advantage of the dissemination aspect of the platform.

Gender and age also plays a role in users’ level of engagement. On the one hand, females are more likely than males to have read most of the items in the library and have stronger intention to read and cite as well. Interestingly, they are less likely not only to add all of their publications to the library but also to add items with the community in mind. On the other hand, the older the respondents are, the less likely they cite items in the library, but the more they only add items of high quality. As they grow older, they are more likely to value the social networking function of the social reference managers, both in keeping contact with other scholars and maintaining current in research, though only slightly.

### Ideology

Most Mendeley and Zotero users exhibit a high degree of openness, reflected in their support for open access, open source software, as well as being early adopters of new technology. About 85% of Mendeley users and 90% of Zotero users consider themselves advocates of open access and open-source software. Although fewer consider themselves early adopters of new technology, this still accounts for nearly 70% of Mendeley and 80% of Zotero users (S22 Table).

**Fig 11:**
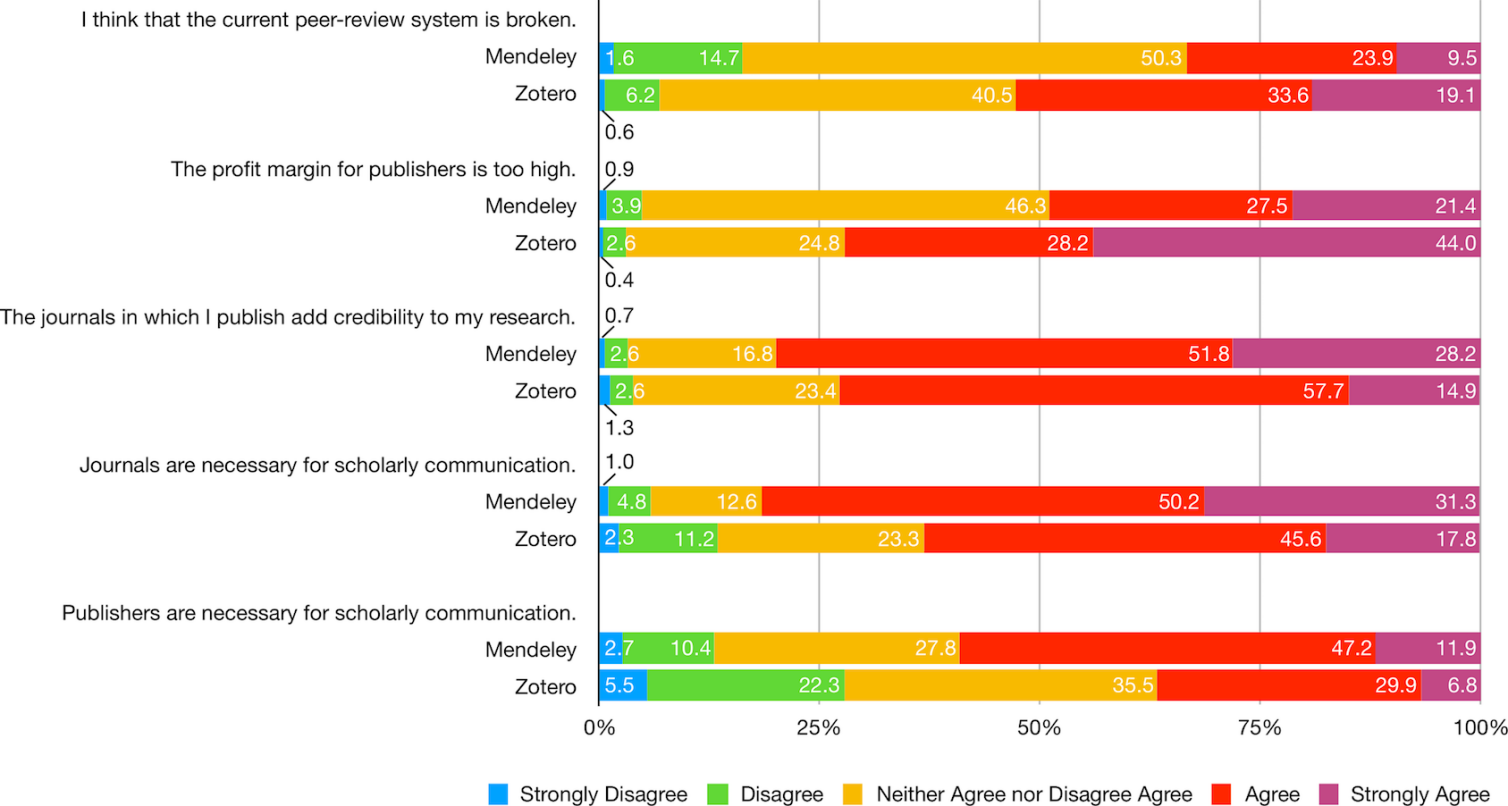
Indicate your level of agreement with the following statements—Peer-review system and scholarly publication.

The ideological cleavage between Mendeley and Zotero users is most clearly seen in their attitudes toward the current journal-based publishing and peer-review system. While 50% of Mendeley and 40% of Zotero users have no strong opinions on whether the current peer-review system is broken, more than 50% of Zotero believe it is whereas only 33% of Mendeley users hold the same opinion. At the same time, 80% of Mendeley and 73% of Zotero users acknowledge that journals add credibility to their research. This is largely in line with their agreement that journals are necessary for scholarly communication, accounting for 82% of Mendeley and 63% of Zotero users. It should be noted, however, that the percentages of Zotero users in support of these two statements are consistently lower compared to Mendeley users. The split between ideological perspectives is manifested even more in their views toward the publisher’s profit margin: 46% of Mendeley users remain in the middle ground, whereas 44% of Zotero users strongly agree that the publisher’s profit margin is too high. More broadly speaking, those who are critical of this phenomenon account for 72% of Zotero users but only 49% of Mendeley users. Consistent with this result, nearly 60% of Mendeley users agree that publishers are necessary for scholarly communication, whereas only 37% of Zotero users do (Fig 11; S23 Table).

**Fig 12:**
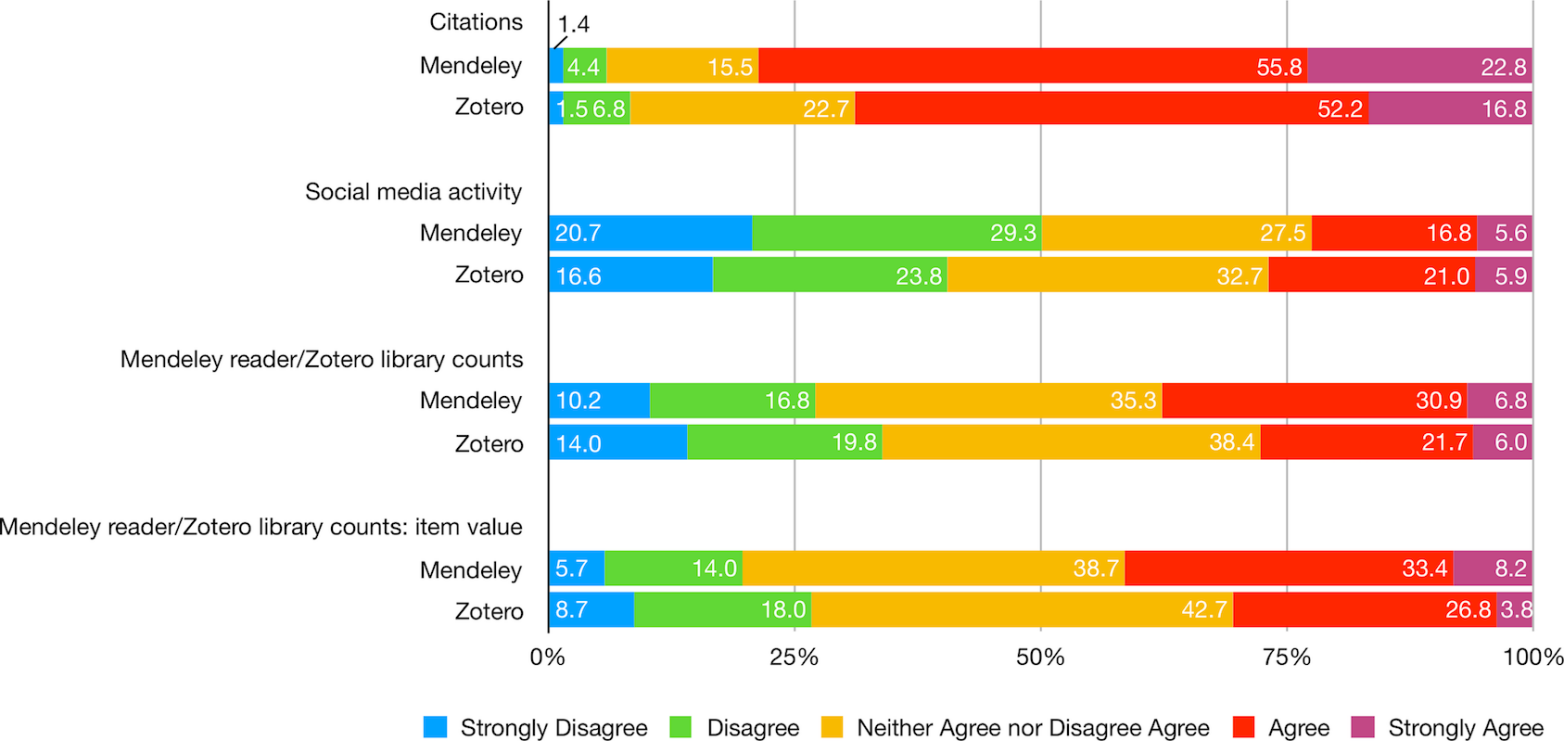
Indicate your level of agreement with the following statements—Indicator of scholarly impact.

Among various indicators, citations are still widely recognized as an appropriate indicator of scholarly impact, as agreed by 79% of Mendeley and 69% of Zotero users. In contrast, respondents’ attitude toward social media activity and altmetrics are more cautious. The majority of Mendeley users and 40% of Zotero users doubt the utility of social media activity, such as tweets and Facebook likes, as indicators of scholarly impact (only a quarter of each support the idea). When asked whether Mendeley readership/Zotero library counts should be used as an indicator of scholarly impact, 37% of all respondents had no strong opinion, while only 38% of Mendeley and 28% of Zotero users were supportive. The idea that the social reference manager-based counts of a document is a good indicator of its value gained similar yet slightly higher support: nearly 40% of all respondents held no strong opinions, and those who support the idea account for 41% of Mendeley users and 31% of Zotero users (Fig 12; S24 Table)

One of the features of social reference managers is the ability to create a profile online, which is assumed to increase the user’s visibility in the academic community. However, more than 40% of Mendeley and Zotero users respectively make no comment on this statement. Notably, a reverse direction of opinions is perceived between Mendeley and Zotero users, where 44% of the Zotero users disagree and 28% of Mendeley users agree that having a social reference manager profile makes them more visible in their field. While it is tempting to conclude that Zotero users may be less concerned about online visibility, 53% of Zotero users consider being visible online critical for their scholar identity, which is 8% higher than the share of Mendeley users. A plausible interpretation is that while respondents care much about their online visibility as part of their scholar identity, social reference manager profiles may not be the prime site for them to construct such an identity and maintain their online visibility. At the same time, over 80% of all respondents agree that maintaining their privacy online is very important to them, and there is no statistically significant difference between platforms. It seems that while online visibility and privacy are both desired, privacy is still the most dominant concern for users in the online environment (S25 Table).

S26–S29 Tables provide regression results related with the ideological aspects of the two platforms. Zotero users in general are strong advocate for open access and early adopters of technology. They are more critical of the current scholarly communication system centered on journals and publishers, thinking that the current peer-review system is broken and the profit margin for publishers too high, and that journals and publishers are not necessary for scholarly communication. They do, nonetheless, similar to Mendeley user, acknowledge the symbolic capital of journal. At the same time, they are also more critical of the use of Mendeley readership/Zotero library counts as a good indicator of scholarly impact, indicating not only their aversion to the traditional model of scholarly communication relying on journal-based publication and peer-review system but also their more conservative view toward metrics generated by social reference managers.

The overall effect of occupation is statistically significant in a broad range of issues related to scholarly communication. Compared to students, non-students (i.e., professors, lecturers, and practitioners) are more likely to identify themselves as an early adopter of new technology. Non-students are also more likely to regard social media activity and Mendeley reader/Zotero library counts as proper indicators of scholarly impact. In addition, lecturers and researchers have more negative perceptions of the role of publishers in scholarly communication, but lecturers are more inclined to contend that having a profile on the social reference managers increase their visibility in their field.

Disciplinary and educational differences are revealing in how altmetrics are perceived. On the one hand, compared to their counterparts in social sciences, respondents affiliated with engineering, physical sciences, and psychology oppose more strongly to the idea of social media activity being used as an indicator of scholarly impact. Physical scientists do not favor the idea that social reference manager-based metrics reflect the value of a document either. On the other hand, those holding a doctorate degree share much of the same negative attitudes toward altmetrics as compared to those with a master’s or professional degree.

Lastly, gender and age also have impacts on ideological positions. Females are less likely than their male counterparts to be the early adopter of new technologies, but more likely to agree that journals and publishers are necessary for scholarly communication. The increase in age tends to have the same impact on evaluating journals and publishers in scholarly communication more positively. Moreover, the older the respondents are, the more likely they are to think that having a profile on social reference managers would increase their visibility in their field.

## Discussion

### The portrait of users

Altmetrics are increasingly used as indicators of attention given to scholarly documents. It is therefore of critical importance to have a better understanding of the audience generating these metrics. This paper first describes the characteristics of the users of the two platforms and shows that those differ significantly. This suggests that solely relying on Mendeley-based indicators—for example in the Altmetric Attention Score—only provides a partial portrait of readership behavior and cannot be generalized to the broader population of social reference managers. More specifically, Zotero’s userbase is male-skewed and slightly older than Mendeley users. Zotero users are highly loyal in terms of both length and frequency of use. Nearly a quarter identify as practitioners whereas less than 10% of Mendeley users indicate the same occupational type. The plurality of Zotero users report education in the arts and humanities and tend to be more collaborative in their use of the platform than their Mendeley counterparts. They are also more socially-engaged online: making greater use of social sites (e.g., Facebook, Wikipedia, Twitter, Reddit) as well as blogging and repository engagement. Mendeley users, on the other hand, are more gender-balanced and slightly younger. They are fickle in their use and more likely to employ other types of reference management systems (e.g., EndNote). They cater to the life and social sciences. However, some aspects were consistent across both groups: most users were students, researchers, or professors and about 90% of users (across platforms) held a graduate degree or above, which is in line with previous studies of Mendeley user profiles [24,25,36]. Although this might sound obvious—social reference managers are specific tools that have little use to those not dealing with scholarly documents—the finding refutes the claim that social reference managers are indicative of the broader social impact of scholarship. At best, readership counts can be seen as a broader indicator of scholarly impact—i.e., impact on students—although the high correlations with citations [29] suggest that students’ reading patterns align with those of other scholarly practices.

### The portrait of use

Altmetrics focus on the interaction that users exhibit with objects online. To fully understand these interactions, we examined the ways in which both Mendeley and Zotero users interacted with the platform and, thereby, the scholarly objects. As with their demographic information, Mendeley and Zotero users differed in the use of the platform. Zotero users were more active overall, more frequently engaging in tasks such as using search functions, adding to, deleting from, and organizing their libraries. They were also more likely to upload other types of data, such as datasets, figures, or videos and to embed references using the cite function or create bibliographies. Their high activity levels were matched with the comparatively higher importance they placed on these features. Mendeley users engage in some of discovery and networking functions relatively more frequently—such as browsing papers and groups and updating profile information. This could be an artifact of the different age and occupational stage of users—with Mendeley having proportionally more students and on, average, younger users.

Resonating with the stated utility of the platforms, their importance in writing the research project and contributing to the construction of a thesis are shared by both Mendeley and Zotero users. Other than that, however, the platform were exploited in rather different ways, with Zotero not only highly integrated in the research project but also utilized for educational (e.g., course teaching and course assignment) and professional purposes. These differences are not trivial: when interpreting the impact of a work through altmetric indicators, the intended function—or group of users it covers—is foundational, as indicators must be associated to a corresponding concept. This again reinforces the heterogeneity of altmetrics, even when focusing exclusively on the subset of readership metrics.

### Deep impact

The word impact is often bandied about carelessly among scientometricians. However, at the risk of sounding pedantic, we return to Merriam-Webster, which defines impact as having a “direct effect upon” or to “to strike forcefully.” We seek, therefore, to understand the level of effect that readership counts measures. Our results suggest that these indicators may not be as “forceful” as imagined. For example, only one fifth of users (from both platforms) report that they have read the last item that they added to their libraries. The majority of Zotero users state that they “intend to read” the majority of items currently in their libraries. The users themselves also do not see the tool as a signaling device: they tend not to consider quality when adding an item and do not consider the community aspects of saving items. Lecturers and practitioners are the exceptions, who tend to think about the community aspects of saving items. This is likely an artifact of the use for pedagogy and practice.

Both sets of users functionally used the platforms throughout the process of preparing a manuscript; the differences arose in their use of the platforms for post-publication dissemination. Mendeley users were far more likely to consider the platform as a useful dissemination vehicle compared to Zotero users. One could consider this activity as the equivalent to self-citing—by generating a metric for their own work. However, there were gender differences, with females more likely to have read and have intentions to cite the items in their libraries than their male counterparts. This is consistent with the literature on patterns of self-citing, suggesting that these gendered patterns of self-promotion carry into this online space. These results suggest that caution should be used when interpreting usage data from social reference managers as impact indicators.

### Platforming ideologies

Openness is a pervasive ideology across platforms—the large majority of both Mendeley and Zotero users report high levels of support for open access and open source software, though Zotero users are statistically more likely to be strong advocates for open access. Platform differences become apparent when examining the system of peer-review and journal publishing, with a more pessimistic view on the *status quo* from Zotero users. More than half of Zotero users reported that the current peer review system is broken, whereas only a third of Mendeley users reported the same. Fewer Zotero than Mendeley users thought that journals added credibility or were necessary for scholarly communication. Publishers received the lowest reviews from Zotero users in terms of both their capacity and profit margins.

Across both platforms, citations were most likely to be seen as useful indicators of scholarly impact than social media, generally, or readership counts specifically. However, there were platform differences in this, with Zotero users rated lower the use of readership metrics as indicators of scholarly impact and were less likely to consider readership counts indicative of the value of an item. However, neutral responses were the plurality for both platform—suggesting a high degree of ambiguity regarding the current utility of altmetrics as indicators of scholarly impact.

Controlling for platform, differences in ideology emerge on various demographic characteristics. Students, perhaps counterintuitively, are less likely to identify as early adopters of technology or to consider social media activity as an indicator of scholarly impact. Women are less likely to consider themselves as early adopters. Women are also report more positive views on journals and publishers—a view shared with older respondents. Practitioners are more likely to see readership counts as useful indicators of scholarly impacts. Physical scientists do not view readership counts as indicating the value of a document and those in engineering, physical sciences, and psychology are much more cautious about the use of social media activity as an indicator of scholarly impact than their colleagues in the social sciences. Those with a doctoral degree are much more likely than those with a master’s or professional degree to discount the value or use of social media indicators and readership metrics as indicators of scholarly impact. The significant differences in ideology call into question a universal interpretation of the meanings of altmetrics: if those generating the metrics vary in the value they ascribe to the action, those generating indicators should be cautious in ascribing a standardized concept to the metric.

## Conclusion

Readership metrics are generated by a variety of different types of users and these users bring competing practices and ideologies to the mix. One of the only unifying characteristics of those using social reference managers is that they are highly educated. This suggests that Mendeley readership counts should not be seen as an indicator of social impact, but rather to represent another dimension of scholarly impact. However, the functional use of the platforms varied significantly both between the platforms and by various user groups within the platform. Readership counts, therefore, should not be seen as monolithic, but represent various concepts to different user communities. These communities also varied considerably in their ideologies toward scholarly communication and the values they ascribed to metrics. These competing value systems and general ambiguity towards social media metrics should urge caution around the inclusion of readership metrics for those in the business of making and using altmetric indicators.

## Supporting information

S1 File Survey instrument. (DOCX)

S1 Table Comparison of Mendeley user profiles. (PDF)

S2 Table How long have you been using Mendeley/Zotero? (PDF)

S3 Table Which best describes your use of Mendeley/Zotero over time? (PDF)

S4 Table Are you part of any private or public groups on Mendeley/Zotero? (PDF)

S5 Table Tools for professional purposes (i.e., those associated with your vocation rather than for personal use). (PDF)

S6 Table How often do you do the following discoverability tasks on Mendeley/Zotero? (PDF)

S7 Table How often do you do the following adding and organizing tasks on Mendeley/Zotero? (PDF)

S8 Table How often do you do the following re-using task on Mendeley? (PDF)

S9 Table How often do you do the following networking tasks on Mendeley/Zotero? (PDF)

S10 Table Please rate the importance of the following types of features. (PDF)

S11 Table Logistic regression on rating the importance of the following types of features. (PDF)

S12 Table How important is Mendeley/Zotero to you at various stages of a research project? (PDF)

S13 Table Logistic regression on rating the importance of Mendeley/Zotero at various stages of a research project. (PDF)

S14 Table My Mendeley/Zotero library has contributed to my: (select all that apply). (PDF)

S15 Table Logistic regression on the Mendeley/Zotero activities. (PDF)

S16 Table Consider the last item you remember having added to your library. Which of these statements best characterizes your level of engagement with it?
(PDF)

S17 Table Consider the last item you remember having added to your library. Which of these statements best characterize your citation behavior?
(PDF)

S18 Table Indicate your level of agreement with the following statements. (PDF)

S19 Table Logistic regression on the level of engagement—Reading and citation behavior. (PDF)

S20 Table Logistic regression on the level of engagement—Curation behavior. (PDF)

S21 Table Logistic regression on the level of engagement—Social behavior. (PDF)

S22 Table Indicate your level of agreement with the following statements—Openness. (PDF)

S23 Table Indicate your level of agreement with the following statements—Peer-review system and scholarly publication. (PDF)

S24 Table Indicate your level of agreement with the following statements—Indicator of scholarly impact. (PDF)

S25 Table Indicate your level of agreement with the following statements—Online privacy, visibility, and scholarly identity. (PDF)

S26 Table Logistic regression on ideology—Openness. (PDF)

S27 Table Logistic regression on ideology—Peer-review system and scholarly publishing. (PDF)

S28 Table Logistic regression on ideology—Proper indicator of scholarly impact. (PDF)

S29 Table Logistic regression on ideology—Online privacy, visibility, and scholarly identity. (PDF)
