# Correction: Social reference managers and their users: A survey of demographics and ideologies

**DOI:** 10.1371/journal.pone.0202315

**Published:** 2018-08-09

**Authors:** Pei-Ying Chen, Erica Hayes, Vincent Larivière, Cassidy R. Sugimoto

There is an error in affiliation 2 for author Erica Hayes. Affiliation 2 should be: North Carolina State University Libraries, Raleigh, North Carolina, United States of America.
