# Supplementary material for: Social reference managers and their users: A survey of demographics and ideologies
Source: PLoS One. 2018 Jul 11;13(7):e0198033. doi: 10.1371/journal.pone.0198033 (PMC6040870; doi:10.1371/journal.pone.0198033)
Supplement: S1 File — (DOCX) [file pone.0198033.s001.docx]

Survey Instrument

How long have you been using Mendeley/Zotero?

- <3 months
- 3 to <6 months
- 6 months to <1 year
- 1 to < 4 years
- 4 to < 7 years
- > 7 years^[[1]](#footnote-1)^
- Not sure

Which Mendeley/Zotero platform do you primarily use?^[[2]](#footnote-2)^

- Online / Zotero for Firefox
- Desktop / Zotero Standalone
- I use them equally
- I am not sure

Which best describes your use of Mendeley/Zotero over time?

- My use has consistently increased.
- My use has consistently decreased.
- My use fluctuates.
- My use has been relatively constant.

Are you part of any private or public groups on Mendeley/Zotero?

- Private only
- Public only
- Both private and public
- None

How often do you do the following discoverability tasks on Mendeley/Zotero?

|  | Daily | Weekly | Monthly | Yearly | <1 year | Never |
| --- | --- | --- | --- | --- | --- | --- |
| Search for items via tags or groups | ○ | ○ | ○ | ○ | ○ | ○ |
| Search for items using search function | ○ | ○ | ○ | ○ | ○ | ○ |
| Browse most read papers | ○ | ○ | ○ | ○ | ○ | ○ |

How often do you do the following adding and organizing tasks on Mendeley?

|  | Daily | Weekly | Monthly | Yearly | <1 year | Never |
| --- | --- | --- | --- | --- | --- | --- |
| Upload publications | ○ | ○ | ○ | ○ | ○ | ○ |
| Upload other material (e.g., datasets, figures, videos) | ○ | ○ | ○ | ○ | ○ | ○ |
| Complete or correct the bibliographic information of items | ○ | ○ | ○ | ○ | ○ | ○ |
| Organize items by topic or project (e.g., tags, folders) | ○ | ○ | ○ | ○ | ○ | ○ |
| Annotate items (e.g., add notes, highlights) | ○ | ○ | ○ | ○ | ○ | ○ |
| Delete items from library | ○ | ○ | ○ | ○ | ○ | ○ |

How often do you do the following re-using task on Mendeley?

|  | Daily | Weekly | Monthly | Yearly | <1 year | Never |
| --- | --- | --- | --- | --- | --- | --- |
| Embed references (using cite function or create bibliographies) | ○ | ○ | ○ | ○ | ○ | ○ |

How often do you do the following networking tasks on Mendeley?

|  | Daily | Weekly | Monthly | Yearly | <1 year | Never |
| --- | --- | --- | --- | --- | --- | --- |
| Update profile information | ○ | ○ | ○ | ○ | ○ | ○ |
| Search for or browse users/groups | ○ | ○ | ○ | ○ | ○ | ○ |
| Connect with other users | ○ | ○ | ○ | ○ | ○ | ○ |
| Ask or answer questions on message boards or forums | ○ | ○ | ○ | ○ | ○ | ○ |

Please rate the importance of the following types of features.

|  | Not at all Important | Unimportant | Neither Important nor Unimportant | Important | Extremely Important |
| --- | --- | --- | --- | --- | --- |
| Discoverability (e.g., searching and browsing library, catalog, or Mendeley Suggest^[[3]](#footnote-3)^) | ○ | ○ | ○ | ○ | ○ |
| Adding and organizing (e.g., uploading and annotating) | ○ | ○ | ○ | ○ | ○ |
| Re-using (e.g., embedding references) | ○ | ○ | ○ | ○ | ○ |
| Networking (e.g., sharing and messaging) | ○ | ○ | ○ | ○ | ○ |

How important is Mendeley to you at various stages of a research project?

|  | Not at all Important | Unimportant | Neither Important nor Unimportant | Important | Extremely Important |
| --- | --- | --- | --- | --- | --- |
| Starting a new project (e.g., research or assignment) | ○ | ○ | ○ | ○ | ○ |
| Performing the project (e.g., data collection, analysis) | ○ | ○ | ○ | ○ | ○ |
| Writing (e.g., assignment, paper or thesis) | ○ | ○ | ○ | ○ | ○ |
| Preparing the manuscript for submission (at the end of the writing process) | ○ | ○ | ○ | ○ | ○ |
| Disseminating my manuscript (post publication) | ○ | ○ | ○ | ○ | ○ |

My Mendeley library has contributed to my: (select all that apply)

- Published research
- Thesis/dissertation
- Course teaching
- Course assignments
- Professional practice
- Other practices (please specify)

Indicate your level of agreement with the following statements.

|  | Strongly Disagree | Disagree | Neither Agree nor Disagree | Agree | Strongly Agree |
| --- | --- | --- | --- | --- | --- |
| I am an advocate for open access. | ○ | ○ | ○ | ○ | ○ |
| I am an early adopter of new technologies. | ○ | ○ | ○ | ○ | ○ |
| I am an advocate for open source software. | ○ | ○ | ○ | ○ | ○ |
| Maintaining my privacy online is very important to me. | ○ | ○ | ○ | ○ | ○ |
| I think that the current peer-review system is broken. | ○ | ○ | ○ | ○ | ○ |
| Social media activity (e.g., tweets, Facebook likes) should be used as an indicator of scholarly impact. | ○ | ○ | ○ | ○ | ○ |
| Mendeley reader counts should be used as an indicator of scholarly impact. | ○ | ○ | ○ | ○ | ○ |
| The profit margin for publishers is too high. | ○ | ○ | ○ | ○ | ○ |
| Citations should be used as an indicator of scholarly impact. | ○ | ○ | ○ | ○ | ○ |
| The journals in which I publish add credibility to my research. | ○ | ○ | ○ | ○ | ○ |
| Journals are necessary for scholarly communication. | ○ | ○ | ○ | ○ | ○ |
| The number of Mendeley readers of a document is a good indicator of the value of that item. | ○ | ○ | ○ | ○ | ○ |
| Publishers are necessary for scholarly communication. | ○ | ○ | ○ | ○ | ○ |
| Having a profile on Mendeley makes me more visible in my field. | ○ | ○ | ○ | ○ | ○ |
| Being visible online is critical for my scholarly identity. | ○ | ○ | ○ | ○ | ○ |

Indicate your level of agreement with the following statements.

|  | Strongly Disagree | Disagree | Neither Agree nor Disagree | Agree | Strongly Agree |
| --- | --- | --- | --- | --- | --- |
| I have read most of the items in my Mendeley library. | ○ | ○ | ○ | ○ | ○ |
| I intend to read the items that I add to my Mendeley library. | ○ | ○ | ○ | ○ | ○ |
| I have cited most of the items in my Mendeley library. | ○ | ○ | ○ | ○ | ○ |
| I intend to cite the items that I add to my Mendeley library. | ○ | ○ | ○ | ○ | ○ |
| I add all of my own publications to my Mendeley library. | ○ | ○ | ○ | ○ | ○ |
| I add items to my library to show others that I am aware of the item. | ○ | ○ | ○ | ○ | ○ |
| I only add items of high quality to my library. | ○ | ○ | ○ | ○ | ○ |
| I delete items of low quality from my library. | ○ | ○ | ○ | ○ | ○ |
| Adding items to my Mendeley library is good for the community. | ○ | ○ | ○ | ○ | ○ |
| Mendeley allows me to stay in touch with other scholars in the field. | ○ | ○ | ○ | ○ | ○ |
| Mendeley allows me to keep up to date with current research in the field. | ○ | ○ | ○ | ○ | ○ |

Consider the last item you remember having added to your library. Which of these statements best characterizes your level of engagement with it?

- I have viewed the title and abstract of the item.
- I intend to read the item.
- I have partly read the full text of the item.
- I have read the entire full text of the item.
- I have not, nor do I intend to read any part of the item.

Consider the last item you remember having added to your library. Which of these statements best characterize your citation behavior?

- I have no intention of citing the item.
- I am not sure if I will cite the item.
- I intend to cite the item.
- I cited the item.

Do you use any of the following tools for professional purposes (i.e., those associated with your vocation rather than for personal use)? (select all that apply)

- Other social reference managers (e.g., BibSonomy, CiteULike, Zotero/Mendeley)
- Other non-social reference managers (e.g., EndNote, JabRef)
- Non-academic social networking sites (e.g., Facebook, LinkedIn)
- Academic social networking sites (e.g., Academia.edu, Loop, ResearchGate)
- Publication and citation profiles (e.g., Google Scholar Citation, ResearcherID, ORCID)
- Publication repositories (e.g., arXiv, SSRN, institutional repository)
- Data and code repositories and content re-use sites (e.g., Dryad, Github, Figshare SlideShare)
- Social recommending, rating and reviewing (e.g., F1000Prime, Pubpeer, Reddit)
- Blogging (e.g., ResearchBlogging.org)
- Microblogging (e.g., Twitter, Weibo)
- Wikis (e.g., Wikipedia)
- None of the above

What is your highest degree or level of education?

- High school graduate (or equivalent)
- Bachelor’s degree (or equivalent)
- Master’s or professional degree (or equivalent)
- Doctorate (or equivalent)

Which of the following best characterizes the discipline in which you currently work?

- Arts and Humanities (e.g., Arts, History, Literature, Philosophy)
- Computer Sciences
- Engineering (e.g., Chemical, Civil, Electrical, or Mechanical Engineering)
- Environmental Sciences (e.g., Atmospheric, Earth, or Oceanography Sciences)
- Life Sciences (e.g., Agricultural, Biological, or Medical Sciences)
- Mathematical Sciences
- Physical Sciences (e.g., Astronomy, Chemistry, Physics)
- Psychology
- Social Sciences (e.g., Sociology, Political Sciences, Economics)
- I am not affiliated with a discipline.
- Others (please specify)

Which best characterizes your occupation or status?

- Professor (i.e., research/teaching position) (e.g., assistant/associate/full)
- Lecturer (i.e., teaching position)
- Researcher (e.g., research staff, postdoc)
- Practitioner (e.g., librarian, nurse, physician)
- Student (e.g., bachelor, master, doctoral student)
- None of the above

What is your age?

- A slider to indicate age on a scale from 0 to 100.

What is your gender?

- Male
- Female
- Other
- Prefer not to answer

In which country do you currently reside?

- A list of countries from Afghanistan to Zimbabwe; Other

1. The option is not available in the survey for Mendeley users. [↑](#footnote-ref-1)
2. Due to the different platforms offered by Mendeley and Zotero, wordings of the first two options are rendered differently. [↑](#footnote-ref-2)
3. Mendeley Suggest is not included in the Zotero survey. [↑](#footnote-ref-3)
