# Supplementary material for: Social reference managers and their users: A survey of demographics and ideologies
Source: PLoS One. 2018 Jul 11;13(7):e0198033. doi: 10.1371/journal.pone.0198033 (PMC6040870; doi:10.1371/journal.pone.0198033)
Supplement: S1 Table — (PDF) [file pone.0198033.s002.pdf]

Table S1. Comparison of Mendeley user profiles.

	This study (n = 796)	Users in Mendeley directory [a]	Mohammadi, Thelwall, & Kousha, 2016 (n = 864)	Jeng, He, & Jiang, 2015 (n = 7366)	Haustein & Larivière, 2014	Zahedi, Costas, & Wouters, 2014
Occupation						
Student	34.7%		31%	33.1%	55.6%	57%
Researcher	24.9%		26%	10.8%	22.8%	25%
Professor	20.9%		38% [b]	24.1%	13.1%	9%
Lecturer	6.0%					3%
Practitioner	9.6%		6%	2.3%	8.4%	6%
None of the above	3.9%			29.7% [c]		20%
Broad discipline [d]						
Basic science	15.8%	23.2%	27%			
Social sciences	27.9%	26.5%	27%			
Engineering	11.0%	10.2%	26%			
Medical sciences	27.0%	26.0%	14%			
Arts and humanities	8.1%	7.6%	6%			

Note:

[a] The information is based on the appendix in Mohammadi et al. (2016)'s study.

[b] The study did not distinguish between professorship and lectureship.

[c] The original category in the study is "other research professional," referred to as "position directly denoted research-oriented, but cannot be categorized." Given the combination of research and profession, it is possible that some may be categorized as "researcher"

and some “practitioner,” which may also account for the relatively low percentage of these two categories compared with the other two survey studies.

[d] The broad discipline is grouped according to Mohammadi et al. (2016)’s mapping of research discipline in Mendeley directory: (1) Basic Science: environmental sciences, chemistry, computer and information science, earth science, materials science, mathematics, and physics and astronomy; (2) Social Sciences: business administration, economics, education, management science, psychology, social-sciences, and sports and recreation; (3) Engineering: electrical and electronic engineering as well as engineering broadly defined; (4) Medical Sciences: biological sciences and medicine; (5) Arts and Humanities: arts and literature, humanities, law, linguistics, design, and philosophy.
