# Supplementary material for: Social reference managers and their users: A survey of demographics and ideologies
Source: PLoS One. 2018 Jul 11;13(7):e0198033. doi: 10.1371/journal.pone.0198033 (PMC6040870; doi:10.1371/journal.pone.0198033)
Supplement: S2 Table — (PDF) [file pone.0198033.s003.pdf]

Table S2. How long have you been using Mendeley/Zotero?

Platform	<3 months	3–6 months	6 months to 1 year	1–4 years	4–7 years	7+ years	Not sure
Mendeley	14 (1.8%)	24 (3.1%)	169 (22.0%)	475 (61.9%)	75 (9.8%)	–	10 (1.3%)
Zotero	11 (2.1%)	23 (4.3%)	21 (4.0%)	185 (35.0%)	173 (32.7%)	113 (21.4%)	3 (0.6%)
