# Supplementary material for: Social reference managers and their users: A survey of demographics and ideologies
Source: PLoS One. 2018 Jul 11;13(7):e0198033. doi: 10.1371/journal.pone.0198033 (PMC6040870; doi:10.1371/journal.pone.0198033)
Supplement: S3 Table — (PDF) [file pone.0198033.s004.pdf]

Table S3. Which best describes your use of Mendeley/Zotero over time?

| Platform | Constantly increased | Constantly decreased | Fluctuates | Relatively constant |
|---|---|---|---|---|
| Mendeley | 220 (28.7%) | 58 (7.6%) | 301 (39.2%) | 188 (24.5%) |
| Zotero | 184 (34.8%) | 21 (4.0%) | 138 (26.1%) | 186 (35.2%) |
