# Supplementary material for: Social reference managers and their users: A survey of demographics and ideologies
Source: PLoS One. 2018 Jul 11;13(7):e0198033. doi: 10.1371/journal.pone.0198033 (PMC6040870; doi:10.1371/journal.pone.0198033)
Supplement: S4 Table — (PDF) [file pone.0198033.s005.pdf]

Table S4. Are you part of any private or public groups on Mendeley/Zotero?

Platform	Private only	Public only	Both	None
Mendeley	255<br>(33.2%)	40<br>(5.2%)	89<br>(11.6%)	383<br>(49.9%)
Zotero	201<br>(38.0%)	29<br>(5.5%)	112<br>(21.2%)	187<br>(35.3%)
