# Supplementary material for: Social reference managers and their users: A survey of demographics and ideologies
Source: PLoS One. 2018 Jul 11;13(7):e0198033. doi: 10.1371/journal.pone.0198033 (PMC6040870; doi:10.1371/journal.pone.0198033)
Supplement: S5 Table — (PDF) [file pone.0198033.s006.pdf]

Table S5. Tools for professional purposes (i.e., those associated with your vocation rather than for personal use).

Tools	Mendeley	Zotero	Total
Publication and citation profiles (e.g., Google Scholar Citation, ResearcherID, ORCID)	500 (65.2%)	318 (60.1%)	818 (63.1%)
Academic social networking sites (e.g., Academia.edu, Loop, ResearchGate)	486 (63.4%)	300 (56.7%)	786 (60.6%)
Non-academic social networking sites (e.g., Facebook, LinkedIn)	444 (57.9%)	336 (63.5%)	780 (60.2%)
Wikis (e.g., Wikipedia)	269 (35.1%)	251 (47.4%)	520 (40.1%)
Microblogging (e.g., Twitter, Weibo)	143 (18.6%)	272 (51.4%)	415 (32.0%)
Publication repositories (e.g., arXiv, SSRN, institutional repository)	163 (21.3%)	227 (42.9%)	390 (30.1%)
Data and code repositories and content re-use sites (e.g., Dryad, Github, Figshare SlideShare)	109 (14.2%)	180 (34.0%)	289 (22.3%)
Other non-social reference managers (e.g., EndNote, JabRef)	183 (23.9%)	103 (19.5%)	286 (22.1%)
Blogging (e.g., ResearchBlogging.org)	78 (10.2%)	150 (28.4%)	228 (17.6%)
Other social reference managers (e.g., BibSonomy, CiteULike, Mendeley)	96 (12.5%)	75 (14.2%)	171 (13.2%)
Social recommending, rating and reviewing (e.g., F1000Prime, Pubpeer, Reddit)	45 (5.9%)	50 (9.5%)	95 (7.3%)
None of the above	36 (4.7%)	22 (4.2%)	58 (4.5%)
