# Supplementary material for: Social reference managers and their users: A survey of demographics and ideologies
Source: PLoS One. 2018 Jul 11;13(7):e0198033. doi: 10.1371/journal.pone.0198033 (PMC6040870; doi:10.1371/journal.pone.0198033)
Supplement: S6 Table — (PDF) [file pone.0198033.s007.pdf]

Table S6. How often do you do the following discoverability tasks on Mendeley/Zotero?

Platform	Daily	Weekly	Monthly	Yearly	<1 year	Never
Search for items via tags or groups						
Mendeley	30<br>(3.9%)	82<br>(10.7%)	131<br>(17.1%)	53<br>(6.9%)	55<br>(7.2%)	416<br>(54.2%)
Zotero	57<br>(10.8%)	109<br>(20.6%)	96<br>(18.1%)	38<br>(7.2%)	40<br>(7.6%)	189<br>(35.7%)
Search for items using search function						
Mendeley	96<br>(12.5%)	190<br>(24.8%)	184<br>(24.0%)	61<br>(8.0%)	52<br>(6.8%)	184<br>(24.0%)
Zotero	157<br>(29.7%)	165<br>(31.2%)	64<br>(12.1%)	21<br>(4.0%)	18<br>(3.4%)	104<br>(19.7%)
Browse most read papers						
Mendeley	35<br>(4.6%)	87<br>(11.3%)	125<br>(16.3%)	64<br>(8.3%)	47<br>(6.1%)	409<br>(53.3%)
Zotero	8<br>(1.5%)	42<br>(7.9%)	26<br>(4.9%)	20<br>(3.8%)	26<br>(4.9%)	407<br>(76.9%)
