# Supplementary material for: Social reference managers and their users: A survey of demographics and ideologies
Source: PLoS One. 2018 Jul 11;13(7):e0198033. doi: 10.1371/journal.pone.0198033 (PMC6040870; doi:10.1371/journal.pone.0198033)
Supplement: S7 Table — (PDF) [file pone.0198033.s008.pdf]

Table S7. How often do you do the following adding and organizing tasks on Mendeley/Zotero?

Platform	Daily	Weekly	Monthly	Yearly	<1 year	Never
Upload publications						
Mendeley	63 (8.2%)	253 (33.0%)	216 (28.2%)	58 (7.6%)	36 (4.7%)	141 (18.4%)
Zotero	126 (23.8%)	209 (39.5%)	90 (17.0%)	13 (2.5%)	15 (2.8%)	76 (14.4%)
Upload other material (e.g., datasets, figures, videos)						
Mendeley	12 (1.6%)	52 (6.8%)	87 (11.3%)	49 (6.4%)	56 (7.3%)	511 (66.6%)
Zotero	16 (3.0%)	68 (12.9%)	103 (19.5%)	58 (11.0%)	37 (7.0%)	247 (46.7%)
Complete or correct the bibliographic information of items						
Mendeley	65 (8.5%)	214 (27.9%)	271 (35.3%)	75 (9.8%)	47 (6.1%)	95 (12.4%)
Zotero	84 (15.9%)	202 (38.2%)	156 (29.5%)	35 (6.6%)	19 (3.6%)	33 (6.2%)
Organize items by topic or project (e.g., tags, folders)						
Mendeley	59 (7.7%)	203 (26.5%)	252 (32.9%)	101 (13.2%)	51 (6.6%)	101 (13.2%)
Zotero	100 (18.9%)	191 (36.1%)	158 (29.9%)	42 (7.9%)	18 (3.4%)	20 (3.8%)
Annotate items (e.g., add notes, highlights)						
Mendeley	71 (9.3%)	165 (21.5%)	214 (27.9%)	77 (10.0%)	58 (7.6%)	182 (23.7%)
Zotero	75 (14.2%)	163 (30.8%)	146 (27.6%)	47 (8.9%)	31 (5.9%)	67 (12.7%)
Delete items from library						
Mendeley	19 (2.5%)	68 (8.9%)	199 (25.9%)	167 (21.8%)	101 (13.2%)	213 (27.8%)
Zotero	47 (8.9%)	113 (21.4%)	186 (35.2%)	97 (18.3%)	41 (7.8%)	45 (8.5%)
