# Supplementary material for: Social reference managers and their users: A survey of demographics and ideologies
Source: PLoS One. 2018 Jul 11;13(7):e0198033. doi: 10.1371/journal.pone.0198033 (PMC6040870; doi:10.1371/journal.pone.0198033)
Supplement: S8 Table — (PDF) [file pone.0198033.s009.pdf]

Table S8. How often do you do the following re-using task on Mendeley?

Platform	Daily	Weekly	Monthly	Yearly	< 1 year	Never
Embed references (using cite function or create bibliographies)						
Mendeley	78 (10.2%)	196 (25.6%)	242 (31.6%)	77 (10.0%)	49 (6.4%)	125 (16.3%)
Zotero	95 (18.0%)	187 (35.3%)	148 (28.0%)	38 (7.2%)	16 (3.0%)	45 (8.5%)
