# Supplementary material for: Social reference managers and their users: A survey of demographics and ideologies
Source: PLoS One. 2018 Jul 11;13(7):e0198033. doi: 10.1371/journal.pone.0198033 (PMC6040870; doi:10.1371/journal.pone.0198033)
Supplement: S9 Table — (PDF) [file pone.0198033.s010.pdf]

Table S9. How often do you do the following networking tasks on Mendeley/Zotero?

| Platform | Daily | Weekly | Monthly | Yearly | < 1 year | Never |
|---|---|---|---|---|---|---|
| *Update profile information* | | | | | | |
| Mendeley | 10 (1.3%) | 21 (2.7%) | 57 (7.4%) | 149 (19.4%) | 138 (18.0%) | 392 (51.1%) |
| Zotero | 2 (0.4%) | 8 (1.5%) | 21 (4.0%) | 101 (19.1%) | 141 (26.7%) | 256 (48.4%) |
| *Search for or browse users/groups* | | | | | | |
| Mendeley | 5 (0.7%) | 10 (1.3%) | 62 (8.1%) | 74 (9.6%) | 78 (10.2%) | 538 (70.1%) |
| Zotero | 1 (0.2%) | 7 (1.3%) | 30 (5.7%) | 69 (13.0%) | 103 (19.5%) | 319 (60.3%) |
| *Connect with other users* | | | | | | |
| Mendeley | 2 (0.3%) | 11 (1.4%) | 38 (5.0%) | 76 (9.9%) | 92 (12.0%) | 548 (71.4%) |
| Zotero | 2 (0.4%) | 9 (1.7%) | 26 (4.9%) | 59 (11.2%) | 93 (17.6%) | 340 (64.3%) |
| *Ask or answer questions on message boards or forums* | | | | | | |
| Mendeley | 1 (0.1%) | 8 (1.0%) | 24 (3.1%) | 34 (4.4%) | 44 (5.7%) | 656 (85.5%) |
| Zotero | 1 (0.2%) | 10 (1.9%) | 32 (6.0%) | 75 (14.2%) | 90 (17.0%) | 321 (60.7%) |
