# Supplementary material for: Social reference managers and their users: A survey of demographics and ideologies
Source: PLoS One. 2018 Jul 11;13(7):e0198033. doi: 10.1371/journal.pone.0198033 (PMC6040870; doi:10.1371/journal.pone.0198033)
Supplement: S10 Table — (PDF) [file pone.0198033.s011.pdf]

Table S10. Please rate the importance of the following types of features.

Platform	Extremely unimportant	Unimportant	Neither unimportant or important	Important	Extremely important
Discoverability (e.g., searching and browsing library, catalog, or Mendeley Suggest)					
Mendeley	44 (5.7%)	57 (7.4%)	147 (19.2%)	320 (41.7%)	199 (25.9%)
Zotero	29 (5.5%)	34 (6.4%)	66 (12.5%)	175 (33.1%)	225 (42.5%)
Adding and organizing (e.g., uploading and annotating)					
Mendeley	9 (1.2%)	12 (1.6%)	49 (6.4%)	267 (34.8%)	430 (56.1%)
Zotero	7 (1.3%)	2 (0.4%)	19 (3.6%)	118 (22.3%)	383 (72.4%)
Re-using (e.g., embedding references)					
Mendeley	11 (1.4%)	18 (2.3%)	106 (13.8%)	258 (33.6%)	374 (48.8%)
Zotero	8 (1.5%)	8 (1.5%)	36 (6.8%)	137 (25.9%)	340 (64.3%)
Networking (e.g., sharing and messaging)					
Mendeley	118 (15.4%)	173 (22.6%)	248 (32.3%)	185 (24.1%)	43 (5.6%)
Zotero	87 (16.4%)	135 (25.5%)	165 (31.2%)	122 (23.1%)	20 (3.8%)
