# Supplementary material for: Social reference managers and their users: A survey of demographics and ideologies
Source: PLoS One. 2018 Jul 11;13(7):e0198033. doi: 10.1371/journal.pone.0198033 (PMC6040870; doi:10.1371/journal.pone.0198033)
Supplement: S11 Table — (PDF) [file pone.0198033.s012.pdf]

Table S11. Logistic regression on rating the importance of the following types of features.

	Discoverability (n = 1083)		Adding and organizing (n = 1228)		Re-using (n = 1154)		Networking (n = 883)	
	Coef.	(S.E.)	Coef.	(S.E.)	Coef.	(S.E.)	Coef.	(S.E.)
(Intercept)	1.43***	(0.41)	3.65***	(0.84)	3.92***	(0.69)	-0.87**	(0.33)
Platform								
Zotero	0.23	(0.20)	0.36	(0.44)	0.35	(0.36)	-0.26	(0.16)
Education								
High School	0.33	(0.77)	14.87	(1543.00)	-0.14	(1.08)	2.28**	(0.81)
Bachelor	0.82*	(0.40)	-0.05	(0.67)	0.29	(0.65)	-0.05	(0.27)
Doctorate	-0.03	(0.23)	-0.59	(0.49)	-0.13	(0.40)	-0.60**	(0.19)
X^2	4.6		1.5		0.39		18.4***	
Discipline								
Arts & Humanities	-0.16	(0.30)	0.65	(0.84)	0.24	(0.57)	-0.32	(0.25)
Computer Sciences	0.09	(0.48)	0.28	(1.11)	-0.43	(0.69)	-0.07	(0.36)
Engineering	-0.13	(0.35)	-0.62	(0.63)	0.25	(0.68)	0.30	(0.31)
Environmental Sciences	0.06	(0.52)	0.23	(1.10)	0.48	(1.07)	0.31	(0.39)
Life Sciences	0.10	(0.28)	-0.49	(0.54)	-0.09	(0.46)	0.15	(0.22)
Mathematical Sciences	-0.55	(0.71)	14.84	(1765.84)	-0.95	(1.13)	-0.28	(0.71)
Physical Sciences	-0.11	(0.42)	-0.16	(0.85)	-0.62	(0.63)	0.23	(0.35)
Psychology	-0.04	(0.44)	0.21	(1.10)	-0.42	(0.69)	-0.84*	(0.39)
None	-0.39	(0.62)	-1.02	(1.20)	-1.17	(0.78)	-1.56*	(0.62)
Others	-0.13	(0.31)	1.06	(1.10)	1.81	(1.06)	0.10	(0.26)
X^2	2.1		5.8		9.7		18.2	
Occupation								
Professor	-0.14	(0.30)	1.49*	(0.72)	0.54	(0.60)	0.53*	(0.26)
Lecturer	0.73	(0.50)	1.18	(1.08)	-0.13	(0.69)	0.87**	(0.33)
Researcher	-0.12	(0.26)	0.66	(0.55)	-0.11	(0.49)	0.11	(0.22)
Practitioner	0.22	(0.30)	0.60	(0.66)	-0.25	(0.54)	0.96***	(0.25)
None	-0.42	(0.40)	1.01	(1.11)	-0.33	(0.73)	0.09	(0.39)
X^2	5.3		4.8		2.5		22.5***	
Gender								
Female	-0.22	(0.19)	0.57	(0.43)	0.08	(0.34)	-0.01	(0.16)
Other	-0.27	(0.58)	14.78	(1231.22)	14.08	(812.38)	0.03	(0.54)
X^2	1.5		1.8		0.052		0.0061	
Age	0.01	(0.01)	-0.02	(0.02)	-0.02	(0.02)	0.02*	(0.01)

Note: * $p < 0.05$ ** $p < 0.01$ *** $p < 0.001$ (two-tailed tests)
