# Supplementary material for: Social reference managers and their users: A survey of demographics and ideologies
Source: PLoS One. 2018 Jul 11;13(7):e0198033. doi: 10.1371/journal.pone.0198033 (PMC6040870; doi:10.1371/journal.pone.0198033)
Supplement: S12 Table — (PDF) [file pone.0198033.s013.pdf]

Table S12. How important is Mendeley/Zotero to you at various stages of a research project?

Platform	Extremely unimportant	Unimportant	Neither unimportant or important	Important	Extremely important
Starting a new project (e.g., research or assignment)					
Mendeley	18 (2.3%)	33 (4.3%)	75 (9.8%)	372 (48.5%)	269 (35.1%)
Zotero	3 (0.6%)	14 (2.6%)	34 (6.4%)	190 (35.9%)	288 (54.4%)
Performing the project (e.g., data collection, analysis)					
Mendeley	48 (6.3%)	98 (12.8%)	155 (20.2%)	308 (40.2%)	158 (20.6%)
Zotero	15 (2.8%)	51 (9.6%)	97 (18.3%)	200 (37.8%)	166 (31.4%)
Writing (e.g., assignment, paper or thesis)					
Mendeley	9 (1.2%)	12 (1.6%)	35 (4.6%)	261 (34.0%)	450 (58.7%)
Zotero	3 (0.6%)	6 (1.1%)	27 (5.1%)	143 (27.0%)	350 (66.2%)
Preparing the manuscript for submission (at the end of the writing process)					
Mendeley	19 (2.5%)	38 (5.0%)	62 (8.1%)	250 (32.6%)	398 (51.9%)
Zotero	8 (1.5%)	16 (3.0%)	70 (13.2%)	146 (27.6%)	289 (54.6%)
Disseminating my manuscript (post publication)					
Mendeley	90 (11.7%)	138 (18.0%)	256 (33.4%)	193 (25.2%)	90 (11.7%)
Zotero	113 (21.4%)	141 (26.7%)	177 (33.5%)	62 (11.7%)	36 (6.8%)
