# Supplementary material for: Social reference managers and their users: A survey of demographics and ideologies
Source: PLoS One. 2018 Jul 11;13(7):e0198033. doi: 10.1371/journal.pone.0198033 (PMC6040870; doi:10.1371/journal.pone.0198033)
Supplement: S13 Table — (PDF) [file pone.0198033.s014.pdf]

Table S13. Logistic regression on rating the importance of Mendeley/Zotero at various stages of a research project.

	Starting a new project (n = 1187)	Performing the project (n = 1044)	Writing (n = 1234)	Preparing the manuscript for submission (n = 1164)	Disseminating my manuscript (n = 863)
(Intercept)	2.75***	0.48	4.47***	2.76***	0.18
	(0.60)	(0.37)	(0.87)	(0.53)	(0.34)
Platform					
Zotero	1.01**	0.38*	0.83	0.77**	-1.09***
	(0.33)	(0.19)	(0.46)	(0.29)	(0.17)
Education					
High School	-1.16	0.44	-1.51	-0.15	0.83
	(0.68)	(0.81)	(0.85)	(1.08)	(0.76)
Bachelor	-0.24	0.55	-0.11	-0.44	0.29
	(0.49)	(0.33)	(0.66)	(0.38)	(0.27)
Doctorate	-0.16	-0.84***	0.88	0.26	-0.42*
	(0.34)	(0.22)	(0.55)	(0.33)	(0.20)
X^2	3.0	20.7***	6.8	2.4	8.0*
Discipline					
Arts & Humanities	-0.53	1.25***	-0.07	0.41	0.04
	(0.43)	(0.32)	(0.62)	(0.41)	(0.26)
Computer Sciences	0.4	0.46	14.96	-0.24	0.44
	(0.79)	(0.43)	(881.09)	(0.50)	(0.37)
Engineering	-0.24	1.02**	0.22	0.70	1.33***
	(0.47)	(0.36)	(0.83)	(0.52)	(0.32)
Environmental Sciences	0.39	0.05	0.01	0.73	1.08*
	(0.79)	(0.40)	(1.11)	(0.78)	(0.44)
Life Sciences	0.22	0.26	0.37	0.94*	0.41
	(0.40)	(0.23)	(0.61)	(0.39)	(0.23)
Mathematical Sciences	-0.24	0.90	14.23	-0.19	0.55
	(1.10)	(0.82)	(1,786.45)	(1.09)	(0.66)
Physical Sciences	0.01	0.78	-0.51	0.28	0.55
	(0.60)	(0.42)	(0.85)	(0.58)	(0.35)
Psychology	-0.61	-0.71*	-0.69	-0.09	-0.46
	(0.53)	(0.34)	(0.74)	(0.50)	(0.36)
None	-1.55*	-0.71	-0.75	-0.93	-0.19
	(0.79)	(0.61)	(0.99)	(0.64)	(0.59)
Others	1.13	0.44	0.85	0.70	0.16
	(0.77)	(0.31)	(0.84)	(0.47)	(0.28)
X^2	12.2	37.0***	4.7	14.8	29.9***
Occupation					
Professor	-0.37	0.52	-0.55	-0.06	0.13
	(0.45)	(0.28)	(0.68)	(0.44)	(0.27)
Lecturer	0.57	1.83	-0.27	-0.13	1.09**
	(0.77)	(0.62)	(0.83)	(0.53)	(0.36)
Researcher	-0.64	0.59**	-0.27	-0.11	-0.01

	Starting a new project (n = 1187)	Performing the project (n = 1044)	Writing (n = 1234)	Preparing the manuscript for submission (n = 1164)	Disseminating my manuscript (n = 863)
	(0.39)	(0.25)	(0.64)	(0.39)	(0.23)
Practitioner	0.00	0.81*	-0.88	-0.56	0.34
	(0.51)	(0.30)	(0.59)	(0.38)	(0.25)
None	0.05	0.06**	0.37	-0.59	-0.03
	(0.71)	(0.41)	(1.27)	(0.55)	(0.40)
X^2	4.6	16.4**	3.0	2.9	11.7*
Gender					
Female	0.39	-0.02	0.17	0.23	0.03
	(0.29)	(0.18)	(0.43)	(0.27)	(0.16)
Other	-0.24	-0.70	-2.27***	-1.68**	-1.67*
	(0.79)	(0.56)	(0.68)	(0.56)	(0.81)
X^2	2.2	1.6	13.0**	11.5**	4.4
Age	0.00	0.01	-0.03	-0.02	-0.01
	(0.01)	(0.01)	(0.02)	(0.01)	(0.01)

Notes: The numbers in the parentheses are standard errors.

* $p < 0.05$ ** $p < 0.01$ *** $p < 0.001$ (two-tailed tests)
