# Supplementary material for: Social reference managers and their users: A survey of demographics and ideologies
Source: PLoS One. 2018 Jul 11;13(7):e0198033. doi: 10.1371/journal.pone.0198033 (PMC6040870; doi:10.1371/journal.pone.0198033)
Supplement: S14 Table — (PDF) [file pone.0198033.s015.pdf]

Table S14. My Mendeley/Zotero library has contributed to my: (select all that apply)

Contribution	Mendeley	Zotero	Total
Published research	443<br>(57.8%)	377<br>(71.3%)	820<br>(63.3%)
Thesis/dissertation	451<br>(58.8%)	282<br>(53.3%)	733<br>(56.6%)
Course teaching	158<br>(20.6%)	246<br>(46.5%)	404<br>(31.2%)
Course assignments	204<br>(26.6%)	190<br>(35.9%)	394<br>(30.4%)
Professional practice	286<br>(37.3%)	318<br>(60.1%)	604<br>(46.6%)
Other practices	36<br>(4.7%)	55<br>(10.4%)	91<br>(7.0%)
