# Supplementary material for: Social reference managers and their users: A survey of demographics and ideologies
Source: PLoS One. 2018 Jul 11;13(7):e0198033. doi: 10.1371/journal.pone.0198033 (PMC6040870; doi:10.1371/journal.pone.0198033)
Supplement: S15 Table — (PDF) [file pone.0198033.s016.pdf]

Table S15. Logistic regression on the Mendeley/Zotero activities

	Published research		Thesis/dissertation		Course teaching		Course assignments		Professional practice		Other practices	
	Coef.	(S.E.)	Coef.	(S.E.)	Coef.	(S.E.)	Coef.	(S.E.)	Coef.	(S.E.)	Coef.	(S.E.)
(Intercept)	-0.40	(0.29)	4.14***	(0.35)	-2.34***	(0.32)	-0.06	(0.30)	-2.23***	(0.30)	-4.83***	(0.56)
Platform												
Zotero	0.98***	(0.16)	0.12	(0.15)	1.07***	(0.15)	0.58***	(0.15)	0.73***	(0.14)	0.70**	(0.26)
Education												
High School	-0.71	(0.51)	-0.77	(0.63)	-0.74	(0.68)	-0.69	(0.58)	-0.63	(0.59)	-0.27	(1.08)
Bachelor	-0.84***	(0.23)	-0.54*	(0.27)	-0.22	(0.29)	1.26***	(0.22)	-0.13	(0.24)	0.56	(0.40)
Doctorate	0.92***	(0.17)	-0.24	(0.17)	0.34	(0.17)	-0.39*	(0.18)	-0.22	(0.17)	0.30	(0.30)
X^2	52.7***		5.9		6.7		45.8***		2.7		2.6	
Discipline												
Arts & Humanities	0.09	(0.22)	0.14	(0.23)	0.26	(0.21)	0.10	(0.21)	0.43*	(0.21)	0.08	(0.40)
Computer Sciences	0.87*	(0.35)	0.49	(0.38)	-0.34	(0.37)	-0.28	(0.35)	-0.73*	(0.37)	-0.52	(0.79)
Engineering	0.43	(0.26)	-0.35	(0.28)	-0.65*	(0.31)	-0.31	(0.27)	0.05	(0.26)	-0.09	(0.59)
Environmental Sciences	0.39	(0.37)	0.04	(0.39)	-0.31	(0.40)	-0.84	(0.43)	0.12	(0.35)	0.92	(0.57)
Life Sciences	0.71***	(0.21)	-0.53**	(0.21)	-0.06	(0.21)	-0.13	(0.20)	0.60**	(0.19)	0.50	(0.37)
Mathematical Sciences	1.78	(1.15)	-0.25	(0.64)	-0.02	(0.65)	-0.29	(0.71)	0.26	(0.62)	-13.63	(641.69)
Physical Sciences	0.94*	(0.37)	-1.06**	(0.32)	-0.34	(0.34)	-0.62	(0.36)	-0.34	(0.32)	0.09	(0.67)
Psychology	0.00	(0.33)	0.22	(0.36)	-0.24	(0.37)	0.19	(0.32)	0.32	(0.32)	-0.09	(0.79)
None	-0.52	(0.48)	-1.53*	(0.61)	-0.06	(0.47)	-0.84	(0.54)	0.39	(0.54)	0.78	(0.62)
Others	0.06	(0.23)	-0.45	(0.24)	0.21	(0.23)	0.17	(0.23)	0.68**	(0.24)	0.78*	(0.38)
X^2	25.7**		32.3***		12.0		13.9		29.8***		10.0	
Occupation												
Professor	1.27***	(0.25)	-2.10***	(0.25)	1.29***	(0.24)	-0.12	(0.23)	0.83***	(0.22)	-0.08	(0.43)
Lecturer	0.69*	(0.30)	-1.26***	(0.31)	1.64***	(0.30)	0.32	(0.29)	1.10***	(0.28)	-0.66	(0.78)
Researcher	0.64**	(0.20)	-1.29***	(0.22)	0.35	(0.23)	-0.69**	(0.21)	1.17***	(0.20)	0.19	(0.39)
Practitioner	-0.29	(0.21)	-2.69***	(0.24)	0.80***	(0.23)	-0.25	(0.21)	2.40***	(0.24)	0.56	(0.37)

	Published research		Thesis/dissertation		Course teaching		Course assignments		Professional practice		Other practices	
None	-0.73*	(0.32)	-1.49***	(0.33)	0.24	(0.35)	-0.11	(0.32)	1.09***	(0.31)	0.82	(0.48)
X^2	53.3***		139.9***		54.7***		16.2**		110.9***		7.1	
Gender												
Female	-0.12	(0.14)	-0.15	(0.15)	-0.22	(0.15)	0.11	(0.14)	0.12	(0.14)	-0.06	(0.26)
Other	-0.91*	(0.46)	-0.58	(0.49)	0.20	(0.45)	0.37	(0.43)	0.54	(0.44)	1.40**	(0.52)
X^2	4.3		2.2		2.5		1.2		2.0		7.7*	
Age	0.00	(0.01)	-0.05***	(0.01)	0.01	(0.01)	-0.02**	(0.01)	0.02*	(0.01)	0.03**	(0.01)

Notes: * $p < 0.05$ ** $p < 0.01$ *** $p < 0.001$ (two-tailed tests)
