# Supplementary material for: Social reference managers and their users: A survey of demographics and ideologies
Source: PLoS One. 2018 Jul 11;13(7):e0198033. doi: 10.1371/journal.pone.0198033 (PMC6040870; doi:10.1371/journal.pone.0198033)
Supplement: S16 Table — Which of these statements best characterizes your level of engagement with it? (PDF) [file pone.0198033.s017.pdf]

Table S16. Consider the last item you remember having added to your library. Which of these statements best characterizes your level of engagement with it?

Statement	Mendeley	Zotero	All
I have viewed the title and abstract of the item.	169 (22.0%)	100 (18.9%)	269 (20.8%)
I intend to read the item.	98 (12.8%)	111 (21.0%)	209 (16.1%)
I have partly read the full text of the item.	325 (42.4%)	208 (39.3%)	533 (41.1%)
I have read the entire full text of the item.	172 (22.4%)	105 (19.8%)	277 (21.4%)
I have not, nor do I intend to read any part of the item.	3 (0.4%)	5 (0.9%)	8 (0.6%)
