# Supplementary material for: Social reference managers and their users: A survey of demographics and ideologies
Source: PLoS One. 2018 Jul 11;13(7):e0198033. doi: 10.1371/journal.pone.0198033 (PMC6040870; doi:10.1371/journal.pone.0198033)
Supplement: S17 Table — Which of these statements best characterize your citation behavior? (PDF) [file pone.0198033.s018.pdf]

Table S17. Consider the last item you remember having added to your library. Which of these statements best characterize your citation behavior?

Statement	Mendeley	Zotero	All
I have no intention of citing the item.	36 (4.7%)	35 (6.6%)	71 (5.5%)
I am not sure if I will cite the item.	275 (35.9%)	255 (48.2%)	530 (40.9%)
I intend to cite the item.	293 (38.2%)	172 (32.5%)	465 (35.9%)
I cited the item.	163 (21.3%)	67 (12.7%)	230 (17.7%)
