# Supplementary material for: Social reference managers and their users: A survey of demographics and ideologies
Source: PLoS One. 2018 Jul 11;13(7):e0198033. doi: 10.1371/journal.pone.0198033 (PMC6040870; doi:10.1371/journal.pone.0198033)
Supplement: S18 Table — (PDF) [file pone.0198033.s019.pdf]

Table S18. Indicate your level of agreement with the following statements.

Platform	Strongly Disagree	Disagree	Neither Agree nor Disagree	Agree	Strongly Agree
I have read most of the items in my Mendeley/Zotero library.					
Mendeley	20	114	115	403	115
	(2.6%)	(14.9%)	(15.0%)	(52.5%)	(15.0%)
Zotero	29	152	78	202	68
	(5.5%)	(28.7%)	(14.7%)	(38.2%)	(12.9%)
I intend to read the items that I add to my Mendeley/Zotero library.					
Mendeley	11	40	91	425	200
	(1.4%)	(5.2%)	(11.9%)	(55.4%)	(26.1%)
Zotero	6	55	73	276	119
	(1.1%)	(10.4%)	(13.8%)	(52.2%)	(22.5%)
I have cited most of the items in my Mendeley/Zotero library.					
Mendeley	23	159	154	299	132
	(3.0%)	(20.7%)	(20.1%)	(39.0%)	(17.2%)
Zotero	57	197	114	121	40
	(10.8%)	(37.2%)	(21.6%)	(22.9%)	(7.6%)
I intend to cite the items that I add to my Mendeley/Zotero library.					
Mendeley	12	87	152	355	161
	(1.6%)	(11.3%)	(19.8%)	(46.3%)	(21.0%)
Zotero	28	102	129	194	76
	(5.3%)	(19.3%)	(24.4%)	(36.7%)	(14.4%)
I add all of my own publications to my Mendeley/Zotero library.					
Mendeley	39	143	222	228	135
	(5.1%)	(18.6%)	(28.9%)	(29.7%)	(17.6%)
Zotero	49	100	95	125	160
	(9.3%)	(18.9%)	(18.0%)	(23.6%)	(30.2%)
I only add items of high quality to my library.					
Mendeley	68	226	225	202	46
	(8.9%)	(29.5%)	(29.3%)	(26.3%)	(6.0%)
Zotero	83	196	123	112	15
	(15.7%)	(37.1%)	(23.3%)	(21.2%)	(2.8%)
I delete items of low quality from my library.					
Mendeley	88	237	189	196	57
	(11.5%)	(30.9%)	(24.6%)	(25.6%)	(7.4%)
Zotero	84	170	99	148	28
	(15.9%)	(32.1%)	(18.7%)	(28.0%)	(5.3%)
I add items to my library to show others that I am aware of the item.					
Mendeley	220	233	194	98	22
	(28.7%)	(30.4%)	(25.3%)	(12.8%)	(2.9%)
Zotero	217	181	74	43	14
	(41.0%)	(34.2%)	(14.0%)	(8.1%)	(2.6%)
Adding items to my Mendeley/Zotero library is good for the community.					
Mendeley	61	91	378	190	47
	(8.0%)	(11.9%)	(49.3%)	(24.8%)	(6.1%)
Zotero	73	90	281	67	18
	(13.8%)	(17.0%)	(53.1%)	(12.7%)	(3.4%)
Mendeley/Zotero allows me to stay in touch with other scholars in the field.					
Mendeley	132	162	296	139	38
	(17.2%)	(21.1%)	(38.6%)	(18.1%)	(5.0%)
Zotero	129	163	193	38	6

Platform	Strongly Disagree	Disagree	Neither Agree nor Disagree	Agree	Strongly Agree
	(24.4%)	(30.8%)	(36.5%)	(7.2%)	(1.1%)
Mendeley/Zotero allows me to keep up to date with current research in the field.					
Mendeley	72	106	239	266	84
	(9.4%)	(13.8%)	(31.2%)	(34.7%)	(11.0%)
Zotero	67	115	173	126	48
	(12.7%)	(21.7%)	(32.7%)	(23.8%)	(9.1%)

Notes: * $p < 0.05$ ** $p < 0.01$ *** $p < 0.001$ (two-tailed tests)
