# Supplementary material for: Social reference managers and their users: A survey of demographics and ideologies
Source: PLoS One. 2018 Jul 11;13(7):e0198033. doi: 10.1371/journal.pone.0198033 (PMC6040870; doi:10.1371/journal.pone.0198033)
Supplement: S19 Table — (PDF) [file pone.0198033.s020.pdf]

Table S19. Logistic regression on the level of engagement — Reading and citation behavior.

	I have read most of the items. (n = 1103)		I intend to read the items. (n = 1132)		I have cited most of the items. (n = 1028)		I intend to cite the items. (n = 1015)	
	Coef.	(S.E.)	Coef.	(S.E.)	Coef.	(S.E.)	Coef.	(S.E.)
(Intercept)	1.18***	(0.33)	2.54***	(0.48)	1.86***	(0.32)	2.81***	(0.40)
Platform								
Zotero	-0.74***	(0.16)	-0.31	(0.23)	-1.29***	(0.15)	-0.73***	(0.18)
Education								
High School	0.15	(0.62)	-0.59	(0.70)	1.34	(0.82)	1.77	(1.08)
Bachelor	-0.10	(0.27)	-0.17	(0.39)	0.00	(0.27)	0.26	(0.32)
Doctorate	-0.23	(0.19)	-0.05	(0.28)	-0.12	(0.18)	0.24	(0.21)
X^2	1.7		0.85		3.4		4.0	
Discipline								
Arts & Humanities	0.02	(0.23)	0.10	(0.34)	-0.02	(0.24)	-0.84**	(0.28)
Computer Sciences	0.23	(0.36)	1.24	(0.76)	-0.22	(0.36)	-0.70	(0.41)
Engineering	0.54	(0.31)	0.49	(0.48)	-0.18	(0.28)	-0.92**	(0.34)
Environmental Sciences	0.13	(0.40)	0.77	(0.77)	-0.02	(0.42)	0.16	(0.58)
Life Sciences	0.60**	(0.23)	0.07	(0.32)	-0.34	(0.21)	-0.84**	(0.27)
Mathematical Sciences	-0.24	(0.62)	-0.66	(0.85)	-0.15	(0.77)	-0.68	(0.86)
Physical Sciences	1.19**	(0.42)	1.17	(0.76)	-0.08	(0.35)	-0.65	(0.43)
Psychology	-0.37	(0.35)	-0.15	(0.53)	-0.34	(0.35)	-0.82	(0.42)
None	-0.85	(0.48)	-0.91	(0.57)	-1.18	(0.62)	-1.74***	(0.53)
Others	-0.43	(0.24)	-0.46	(0.34)	-0.11	(0.25)	-0.64*	(0.31)
X^2	29.6**		13.7		6.7		21.0*	
Occupation								
Professor	-0.40	(0.26)	-0.48	(0.37)	-0.15	(0.24)	-0.64*	(0.30)
Lecturer	-0.81*	(0.32)	0.02	(0.58)	-0.52	(0.32)	-0.58	(0.40)
Researcher	-0.71**	(0.23)	-0.43	(0.34)	-0.59**	(0.21)	-0.84**	(0.27)
Practitioner	-0.51*	(0.24)	-0.64	(0.35)	-0.48*	(0.23)	-1.14***	(0.27)
None	-0.46	(0.35)	-0.58	(0.47)	-0.68	(0.36)	-0.91*	(0.38)
X^2	13.2*		4.4		13.0*		20.7***	
Gender								
Female	0.41**	(0.16)	0.68**	(0.24)	0.26	(0.15)	0.51**	(0.18)
Other	0.19	(0.52)	0.33	(0.77)	0.06	(0.52)	0.63	(0.59)
X^2	6.7*		8.2*		2.9		8.3*	
Age	0.01	(0.01)	0.00	(0.01)	-0.02*	(0.01)	-0.01	(0.01)

Notes: * $p < 0.05$ ** $p < 0.01$ *** $p < 0.001$ (two-tailed tests)
