# Supplementary material for: Social reference managers and their users: A survey of demographics and ideologies
Source: PLoS One. 2018 Jul 11;13(7):e0198033. doi: 10.1371/journal.pone.0198033 (PMC6040870; doi:10.1371/journal.pone.0198033)
Supplement: S20 Table — (PDF) [file pone.0198033.s021.pdf]

Table S20. Logistic regression on the level of engagement — Curation behavior.

	I add all of my own publications to my library. (n = 979)		I only add items of high quality to my library. (n = 948)		I delete items of low quality from my library. (n = 1008)	
	Coef.	(S.E.)	Coef.	(S.E.)	Coef.	(S.E.)
(Intercept)	1.18***	(0.34)	-0.84**	(0.32)	0.18	(0.30)
Platform						
Zotero	0.04	(0.17)	-0.67***	(0.16)	-0.32*	(0.15)
Education						
High School	-0.37	(0.64)	-0.09	(0.61)	0.10	(0.56)
Bachelor	-0.44	(0.27)	-0.04	(0.27)	0.19	(0.25)
Doctorate	0.32	(0.19)	-0.36*	(0.18)	-0.35*	(0.18)
X^2	7.0		3.9		5.4	
Discipline						
Arts & Humanities	-0.23	(0.24)	-0.47*	(0.24)	-0.23	(0.22)
Computer Sciences	-0.12	(0.37)	0.62	(0.38)	0.23	(0.36)
Engineering	0.16	(0.30)	-0.13	(0.28)	-0.45	(0.27)
Environmental Sciences	0.90	(0.49)	-0.59	(0.42)	-0.38	(0.37)
Life Sciences	0.77**	(0.24)	-0.37	(0.21)	-0.36	(0.20)
Mathematical Sciences	-0.27	(0.63)	-0.92	(0.82)	-0.98	(0.81)
Physical Sciences	0.54	(0.38)	-0.18	(0.35)	-0.41	(0.35)
Psychology	0.05	(0.37)	-0.12	(0.34)	-0.33	(0.35)
None	-1.67*	(0.68)	-1.18	(0.69)	0.51	(0.53)
Others	0.10	(0.26)	-0.40	(0.26)	0.03	(0.24)
X^2	28.5**		15.5		10.4	
Occupation						
Professor	0.20	(0.26)	-0.12	(0.25)	-0.33	(0.24)
Lecturer	0.63	(0.36)	0.47	(0.32)	0.25	(0.30)
Researcher	0.31	(0.23)	-0.22	(0.22)	-0.39	(0.21)
Practitioner	-0.18	(0.25)	0.08	(0.24)	0.38	(0.22)
None	-0.27	(0.36)	-0.54	(0.40)	-0.43	(0.35)
X^2	7.4		7.2		14.3*	
Gender						
Female	-1.09***	(0.16)	-0.09	(0.15)	-0.28	(0.15)
Other	0.42	(0.56)	-0.58	(0.60)	-0.48	(0.47)
X^2	48.5***		1.2		4.2	
Age	-0.01	(0.01)	0.03***	(0.01)	0.01	(0.01)

Notes: * $p < 0.05$ ** $p < 0.01$ *** $p < 0.001$ (two-tailed tests)
