# Supplementary material for: Social reference managers and their users: A survey of demographics and ideologies
Source: PLoS One. 2018 Jul 11;13(7):e0198033. doi: 10.1371/journal.pone.0198033 (PMC6040870; doi:10.1371/journal.pone.0198033)
Supplement: S21 Table — (PDF) [file pone.0198033.s022.pdf]

Table S21. Logistic regression on the level of engagement — Social behavior.

	I add items to show others that I am aware of the item. (n = 1028)		Adding items to my library is good for the community. (n = 637)		Mendeley/Zotero allows me to stay in touch with other scholars in the field. (n = 807)		Mendeley/Zotero allows me to keep up to date with current research in the field. (n = 884)	
	Coef.	(S.E.)	Coef.	(S.E.)	Coef.	(S.E.)	Coef.	(S.E.)
(Intercept)	-1.73***	(0.40)	1.01*	(0.41)	-1.38***	(0.40)	-0.17	(0.34)
Platform								
Zotero	-0.66**	(0.20)	-1.34***	(0.20)	-1.67***	(0.22)	-0.62***	(0.16)
Education								
High School	1.15	(0.60)	0.72	(0.72)	1.11	(0.74)	0.34	(0.63)
Bachelor	0.02	(0.32)	-0.20	(0.31)	0.24	(0.29)	0.16	(0.28)
Doctorate	-0.47*	(0.23)	-0.36	(0.23)	-0.43	(0.23)	-0.30	(0.19)
X2	9.3*		4.0		7.5		3.9	
Discipline								
Arts & Humanities	-0.06	(0.30)	-0.34	(0.30)	-0.10	(0.31)	-0.03	(0.23)
Computer Sciences	0.59	(0.41)	-0.26	(0.41)	-0.03	(0.42)	0.32	(0.37)
Engineering	0.42	(0.34)	-0.16	(0.35)	0.16	(0.33)	0.15	(0.30)
Environmental Sciences	0.41	(0.46)	0.15	(0.51)	0.40	(0.46)	0.19	(0.39)
Life Sciences	0.28	(0.26)	-0.38	(0.26)	-0.10	(0.25)	0.32	(0.22)
Mathematical Sciences	0.60	(0.83)	-0.90	(0.75)	0.66	(0.91)	0.13	(0.64)
Physical Sciences	-1.00	(0.63)	-1.11*	(0.50)	-0.63	(0.51)	-0.09	(0.38)
Psychology	-0.27	(0.47)	-0.57	(0.43)	-0.12	(0.42)	0.25	(0.36)
None	-0.25	(0.69)	-16.00	(438.52)	-1.59	(1.09)	-0.50	(0.57)
Others	-0.14	(0.33)	0.00	(0.31)	0.38	(0.31)	-0.10	(0.26)
X2	10.8		9.1		8.6		5.4	
Occupation								
Professor	0.09	(0.32)	0.04	(0.31)	0.69*	(0.31)	-0.07	(0.25)
Lecturer	1.46***	(0.34)	1.05*	(0.41)	1.41***	(0.38)	0.77*	(0.36)
Researcher	0.31	(0.27)	-0.33	(0.27)	0.22	(0.28)	-0.04	(0.22)
Practitioner	0.43	(0.29)	0.81*	(0.31)	1.04***	(0.29)	0.09	(0.24)
None	-0.05	(0.46)	0.11	(0.49)	-0.14	(0.51)	-0.33	(0.38)
X2	22.2***		19.1**		25.0***		7.0	
Gender								
Female	-0.30	(0.19)	-0.40*	(0.19)	-0.33	(0.19)	0.15	(0.16)
Other	-0.90	(0.77)	-1.15	(0.59)	-0.83	(0.69)	-0.05	(0.46)
X2	3.6		7.0*		4.0		0.93	
Age	0.01	(0.01)	0.00	(0.01)	0.02*	(0.01)	0.02**	(0.01)

Notes: * $p < 0.05$ ** $p < 0.01$ *** $p < 0.001$ (two-tailed tests)
