# Supplementary material for: Social reference managers and their users: A survey of demographics and ideologies
Source: PLoS One. 2018 Jul 11;13(7):e0198033. doi: 10.1371/journal.pone.0198033 (PMC6040870; doi:10.1371/journal.pone.0198033)
Supplement: S22 Table — (PDF) [file pone.0198033.s023.pdf]

Table S22. Indicate your level of agreement with the following statements — Openness.

Platform	Strongly Disagree	Disagree	Neither Agree nor Disagree	Agree	Strongly Agree
I am an advocate for open access.					
Mendeley	3<br>(0.4%)	22<br>(82.9%)	98<br>(12.8%)	276<br>(36.0%)	368<br>(48.0%)
Zotero	–<br>–	2<br>(0.4%)	48<br>(9.1%)	149<br>(28.2%)	330<br>(62.4%)
I am an early adopter of new technologies.					
Mendeley	13<br>(1.7%)	71<br>(9.3%)	162<br>(21.1%)	361<br>(47.1%)	160<br>(20.9%)
Zotero	6<br>(1.1%)	30<br>(5.7%)	76<br>(14.4%)	264<br>(49.9%)	153<br>(28.9%)
I am an advocate for open source software.					
Mendeley	2<br>(0.3%)	19<br>(2.5%)	109<br>(14.2%)	314<br>(40.9%)	323<br>(42.1%)
Zotero	0<br>(0.0%)	8<br>(1.5%)	53<br>(10.0%)	187<br>(35.3%)	281<br>(53.1%)
