# Supplementary material for: Social reference managers and their users: A survey of demographics and ideologies
Source: PLoS One. 2018 Jul 11;13(7):e0198033. doi: 10.1371/journal.pone.0198033 (PMC6040870; doi:10.1371/journal.pone.0198033)
Supplement: S23 Table — (PDF) [file pone.0198033.s024.pdf]

Table S23. Indicate your level of agreement with the following statements — Peer-review system and scholarly publication.

Platform	Strongly Disagree	Disagree	Neither Agree nor Disagree	Agree	Strongly Agree
I think that the current peer-review system is broken.					
Mendeley	12<br>(1.6%)	113<br>(14.7%)	386<br>(50.3%)	183<br>(23.9%)	73<br>(9.5%)
Zotero	3<br>(0.6%)	33<br>(6.2%)	214<br>(40.5%)	178<br>(33.6%)	101<br>(19.1%)
The profit margin for publishers is too high.					
Mendeley	7<br>(0.9%)	30<br>(3.9%)	355<br>(46.3%)	211<br>(27.5%)	164<br>(21.4%)
Zotero	2<br>(0.4%)	14<br>(2.6%)	131<br>(24.8%)	149<br>(28.2%)	233<br>(44.0%)
The journals in which I publish add credibility to my research.					
Mendeley	5<br>(0.7%)	20<br>(2.6%)	129<br>(16.8%)	397<br>(51.8%)	216<br>(28.2%)
Zotero	7<br>(1.3%)	14<br>(2.6%)	124<br>(23.4%)	305<br>(57.7%)	79<br>(14.9%)
Journals are necessary for scholarly communication.					
Mendeley	8<br>(1.0%)	37<br>(4.8%)	97<br>(12.6%)	385<br>(50.2%)	240<br>(31.3%)
Zotero	12<br>(2.3%)	59<br>(11.2%)	123<br>(23.3%)	241<br>(45.6%)	94<br>(17.8%)
Publishers are necessary for scholarly communication.					
Mendeley	21<br>(2.7%)	80<br>(10.4%)	213<br>(27.8%)	362<br>(47.2%)	91<br>(11.9%)
Zotero	29<br>(5.5%)	118<br>(22.3%)	188<br>(35.5%)	158<br>(29.9%)	36<br>(6.8%)
