# Supplementary material for: Social reference managers and their users: A survey of demographics and ideologies
Source: PLoS One. 2018 Jul 11;13(7):e0198033. doi: 10.1371/journal.pone.0198033 (PMC6040870; doi:10.1371/journal.pone.0198033)
Supplement: S24 Table — (PDF) [file pone.0198033.s025.pdf]

Table S24. Indicate your level of agreement with the following statements — Indicator of scholarly impact.

Platform	Strongly Disagree	Disagree	Neither Agree nor Disagree	Agree	Strongly Agree
Social media activity (e.g., tweets, Facebook likes) should be used as an indicator of scholarly impact.					
Mendeley	159 (20.7%)	225 (29.3%)	211 (27.5%)	129 (16.8%)	43 (5.6%)
Zotero	88 (16.6%)	126 (23.8%)	173 (32.7%)	111 (21.0%)	31 (5.9%)
Mendeley reader/Zotero library counts should be used as an indicator of scholarly impact.					
Mendeley	78 (10.2%)	129 (16.8%)	271 (35.3%)	237 (30.9%)	52 (6.8%)
Zotero	74 (14.0%)	105 (19.8%)	203 (38.4%)	115 (21.7%)	32 (6.0%)
Citations should be used as an indicator of scholarly impact.					
Mendeley	11 (1.4%)	34 (4.4%)	119 (15.5%)	428 (55.8%)	175 (22.8%)
Zotero	8 (1.5%)	36 (6.8%)	120 (22.7%)	276 (52.2%)	89 (16.8%)
The number of Mendeley readers/Zotero libraries of a document is a good indicator of the value of that item.					
Mendeley	44 (5.7%)	107 (14.0%)	297 (38.7%)	256 (33.4%)	63 (8.2%)
Zotero	46 (8.7%)	95 (18.0%)	226 (42.7%)	142 (26.8%)	20 (3.8%)
