# Supplementary material for: Social reference managers and their users: A survey of demographics and ideologies
Source: PLoS One. 2018 Jul 11;13(7):e0198033. doi: 10.1371/journal.pone.0198033 (PMC6040870; doi:10.1371/journal.pone.0198033)
Supplement: S25 Table — (PDF) [file pone.0198033.s026.pdf]

Table S25. Indicate your level of agreement with the following statements — Online privacy, visibility, and scholarly identity.

Platform	Strongly Disagree	Disagree	Neither Agree nor Disagree	Agree	Strongly Agree
Maintaining my privacy online is very important to me.					
Mendeley	5 (0.7%)	30 (3.9%)	110 (14.3%)	283 (36.9%)	339 (44.2%)
Zotero	1 (0.2%)	14 (2.6%)	74 (14.0%)	227 (42.9%)	213 (40.3%)
Having a profile on Mendeley/Zotero makes me more visible in my field.					
Mendeley	78 (10.2%)	144 (18.8%)	331 (43.2%)	171 (22.3%)	43 (5.6%)
Zotero	90 (17.0%)	145 (27.4%)	229 (43.3%)	56 (10.6%)	9 (1.7%)
Being visible online is critical for my scholarly identity.					
Mendeley	52 (6.8%)	102 (13.3%)	270 (35.2%)	238 (31.0%)	105 (13.7%)
Zotero	23 (4.3%)	73 (13.8%)	152 (28.7%)	196 (37.1%)	85 (16.1%)
