# Supplementary material for: Social reference managers and their users: A survey of demographics and ideologies
Source: PLoS One. 2018 Jul 11;13(7):e0198033. doi: 10.1371/journal.pone.0198033 (PMC6040870; doi:10.1371/journal.pone.0198033)
Supplement: S26 Table — (PDF) [file pone.0198033.s027.pdf]

Table S26. Logistic regression on ideology — Openness.

	Advocate for open access (n = 1150)		Early adopter of technology (n = 1058)		Advocate for open source software (n = 1134)	
	Coef.	(S.E.)	Coef.	(S.E.)	Coef.	(S.E.)
(Intercept)	3.54	(0.93)	1.35**	(0.47)	3.93***	(0.89)
Platform						
Zotero	2.01**	(0.76)	0.49*	(0.24)	0.44	(0.47)
Education						
High School	-1.39	(1.14)	0.42	(0.81)	-1.16	(1.10)
Bachelor	0.12	(0.81)	0.51	(0.39)	-0.39	(0.68)
Doctorate	-0.12	(0.51)	-0.22	(0.27)	-0.15	(0.49)
X^2	1.6		3.0		1.3	
Discipline						
Arts & Humanities	1.18	(1.09)	-0.19	(0.34)	0.29	(0.66)
Computer Sciences	0.37	(1.10)	0.57	(0.65)	0.31	(1.09)
Engineering	-0.65	(0.59)	0.57	(0.48)	-0.20	(0.64)
Environmental Sciences	16.02	(1676.05)	0.62	(0.65)	15.41	(1002.42)
Life Sciences	0.24	(0.56)	0.23	(0.31)	0.40	(0.55)
Mathematical Sciences	16.26	(3316.86)	-0.57	(0.86)	-1.16	(1.16)
Physical Sciences	-0.51	(0.74)	-0.13	(0.50)	0.05	(0.83)
Psychology	0.80	(1.09)	-0.68	(0.43)	0.75	(1.09)
None	15.15	(2068.96)	-0.29	(0.83)	15.08	(1370.71)
Others	1.08	(1.09)	-0.15	(0.36)	1.54	(1.09)
X^2	6.3		9.4		4.6	
Occupation						
Professor	-0.41	(0.69)	1.29**	(0.40)	0.30	(0.74)
Lecturer	-0.46	(0.87)	1.37*	(0.63)	0.35	(1.10)
Researcher	-0.20	(0.61)	0.36	(0.29)	-0.81	(0.54)
Practitioner	0.02	(0.86)	0.76*	(0.36)	0.47	(0.84)
None	-0.22	(1.12)	0.18	(0.48)	-0.79	(0.87)
X^2	0.55		15.3**		6.4	
Gender						
Female	0.09	(0.42)	-0.71**	(0.22)	-0.51	(0.42)
Other	14.89	(2114.60)	-0.88	(0.60)	-1.00	(1.10)
X^2	0.05		11.0**		2.0	
Age	-0.01	(0.02)	0.01	(0.01)	0.00	(0.02)

Note: * $p < 0.05$ ** $p < 0.01$ *** $p < 0.001$ (two-tailed tests)
