# Supplementary material for: Social reference managers and their users: A survey of demographics and ideologies
Source: PLoS One. 2018 Jul 11;13(7):e0198033. doi: 10.1371/journal.pone.0198033 (PMC6040870; doi:10.1371/journal.pone.0198033)
Supplement: S27 Table — (PDF) [file pone.0198033.s028.pdf]

Table S27. Logistic regression on ideology — Peer-review system and scholarly publishing.

	Current peer-review system is broken (n = 696)		Profit margin for publishers is too high (n = 810)		Journals where I publish add credibility to my research (n = 1043)		Journals are necessary for scholarly communication (n = 1076)		Publishers are necessary for scholarly communication (n = 895)	
	Coef.	(S.E.)	Coef.	(S.E.)	Coef.	(S.E.)	Coef.	(S.E.)	Coef.	(S.E.)
(Intercept)	1.25**	(0.46)	2.84***	(0.66)	1.94**	(0.71)	0.99*	(0.49)	0.85*	(0.38)
Platform										
Zotero	1.22***	(0.24)	0.80*	(0.34)	-0.05	(0.35)	-1.08***	(0.23)	-1.36***	(0.18)
Education										
High School	-0.71	(0.74)	-0.83	(1.15)	15.39	(1721.01)	-0.48	(0.71)	-0.47	(0.58)
Bachelor	-0.21	(0.38)	0.72	(0.65)	0.05	(0.57)	0.12	(0.37)	0.20	(0.32)
Doctorate	-0.15	(0.26)	0.57	(0.41)	0.31	(0.40)	0.31	(0.27)	0.16	(0.21)
X^2	1.3		3.7		0.59		2.0		1.8	
Discipline										
Arts & Humanities	-0.04	(0.35)	-0.44	(0.45)	0.27	(0.49)	0.27	(0.35)	0.53	(0.28)
Computer Sciences	-0.49	(0.46)	0.02	(0.81)	1.42	(1.06)	-0.74	(0.44)	-0.23	(0.41)
Engineering	-0.09	(0.40)	0.03	(0.61)	0.80	(0.65)	-0.06	(0.42)	0.27	(0.35)
Environmental Sciences	-0.76	(0.49)	-1.27*	(0.61)	0.37	(0.78)	0.41	(0.66)	0.58	(0.51)
Life Sciences	0.17	(0.29)	0.18	(0.49)	0.93	(0.48)	0.07	(0.32)	0.15	(0.25)
Mathematical Sciences	0.21	(0.88)	-1.40	(1.19)	15.51	(1838.51)	13.10	(501.00)	0.43	(1.17)
Physical Sciences	0.36	(0.46)	0.21	(0.80)	0.80	(0.79)	0.12	(0.50)	0.23	(0.39)
Psychology	-0.17	(0.46)	-0.18	(0.70)	0.35	(0.79)	-0.11	(0.58)	-0.60	(0.40)
None	-0.57	(0.86)	14.93	(1424.54)	-1.04	(0.78)	-0.74	(0.67)	0.08	(0.61)
Others	-0.17	(0.37)	0.40	(0.61)	1.18	(0.65)	-0.11	(0.37)	0.05	(0.29)
X^2	6.6		9.1		11.4		6.3		10.1	
Occupation										
Professor	0.26	(0.34)	0.45	(0.51)	1.07	(0.66)	0.17	(0.40)	-0.32	(0.30)
Lecturer	-0.06	(0.44)	0.59	(0.65)	1.23	(1.06)	-0.10	(0.53)	-0.93**	(0.35)
Researcher	0.36	(0.31)	0.44	(0.45)	-0.14	(0.45)	-0.30	(0.31)	-0.91***	(0.25)
Practitioner	1.12**	(0.40)	0.68	(0.53)	-0.27	(0.48)	-0.36	(0.33)	-0.35	(0.28)

	Current peer-review system is broken (n = 696)		Profit margin for publishers is too high (n = 810)		Journals where I publish add credibility to my research (n = 1043)		Journals are necessary for scholarly communication (n = 1076)		Publishers are necessary for scholarly communication (n = 895)	
None	0.66	(0.56)	16.22	(990.14)	0.41	(0.84)	-0.02	(0.55)	0.30	(0.48)
X^2	9.0		2.5		6.2		3.3		20.0**	
Gender										
Female	-0.19	(0.21)	-0.26	(0.32)	0.37	(0.34)	0.71**	(0.25)	0.49**	(0.19)
Other	15.34	(615.01)	-0.43	(0.80)	15.89	(1551.11)	0.58	(0.79)	-0.26	(0.54)
X^2	0.81		0.8		1.2		8.7*		7.5*	
Age	-0.02	(0.01)	-0.03	(0.02)	0.01	(0.02)	0.04**	(0.01)	0.02*	(0.01)

Note: * p < 0.05 ** p < 0.01 *** p < 0.001 (two-tailed tests)
