# Supplementary material for: Social reference managers and their users: A survey of demographics and ideologies
Source: PLoS One. 2018 Jul 11;13(7):e0198033. doi: 10.1371/journal.pone.0198033 (PMC6040870; doi:10.1371/journal.pone.0198033)
Supplement: S28 Table — (PDF) [file pone.0198033.s029.pdf]

Table S28. Logistic regression on ideology — Proper indicator of scholarly impact.

	Social media activity (n = 912)		Mendeley reader/ Zotero library counts (n = 822)		Citations (n = 1057)		Mendeley reader/ Zotero library counts reflect value (n = 773)	
	Coef.	(S.E.)	Coef.	(S.E.)	Coef.	(S.E.)	Coef.	(S.E.)
(Intercept)	-0.09	(0.34)	0.56	(0.35)	2.75***	(0.55)	1.02**	(0.38)
Platform								
Zotero	0.21	(0.17)	-0.59***	(0.17)	-0.37	(0.25)	-0.59***	(0.18)
Education								
High School	0.54	(0.64)	0.32	(0.62)	0.32	(1.07)	1.88	(1.10)
Bachelor	-0.53	(0.29)	0.18	(0.29)	-0.04	(0.44)	-0.14	(0.29)
Doctorate	-0.66***	(0.20)	-0.75***	(0.19)	-0.09	(0.30)	-0.65**	(0.21)
X^2	14.7**		17.7***		0.19		13.5**	
Discipline								
Arts & Humanities	-0.48	(0.25)	-0.30	(0.24)	-0.67	(0.34)	-0.49	(0.26)
Computer Sciences	-0.35	(0.40)	-0.23	(0.39)	0.62	(0.77)	0.12	(0.44)
Engineering	-0.73*	(0.32)	-0.05	(0.30)	-0.10	(0.45)	0.62	(0.36)
Environmental Sciences	-0.78	(0.50)	-0.59	(0.47)	0.12	(0.66)	-0.17	(0.43)
Life Sciences	-0.38	(0.22)	0.13	(0.23)	0.57	(0.40)	-0.06	(0.24)
Mathematical Sciences	-0.25	(0.72)	-0.59	(0.75)	14.07	(721.97)	-0.22	(0.74)
Physical Sciences	-0.75*	(0.38)	-0.58	(0.36)	-0.52	(0.49)	-1.02*	(0.41)
Psychology	-1.07**	(0.41)	-0.46	(0.38)	-0.13	(0.58)	-0.44	(0.40)
None	-0.66	(0.56)	-0.15	(0.61)	-0.99	(0.76)	-1.33*	(0.59)
Others	0.16	(0.26)	0.13	(0.28)	0.31	(0.45)	0.17	(0.29)
X^2	19.0*		9.9		15.0		23.9**	
Occupation								
Professor	0.66*	(0.27)	0.52*	(0.25)	0.19	(0.41)	0.14	(0.27)
Lecturer	0.76*	(0.34)	0.57	(0.34)	0.12	(0.53)	0.62	(0.36)
Researcher	0.41	(0.23)	0.29	(0.23)	-0.29	(0.34)	0.07	(0.24)
Practitioner	1.25***	(0.25)	1.07***	(0.28)	0.34	(0.42)	0.43	(0.28)
None	-0.32	(0.45)	0.11	(0.43)	0.34	(0.63)	0.06	(0.43)
X^2	31.0***		16.7**		3.1		4.9	
Gender								
Female	0.23	(0.16)	0.13	(0.16)	-0.04	(0.25)	-0.16	(0.17)
Other	-0.28	(0.58)	-0.14	(0.49)	-0.53	(0.68)	0.01	(0.63)
X^2	2.5		0.77		0.62		0.9	
Age	-0.02	(0.01)	-0.01	(0.01)	0.00	(0.01)	0.00	(0.01)

Note: * $p < 0.05$ ** $p < 0.01$ *** $p < 0.001$ (two-tailed tests)
