# Supplementary material for: Social reference managers and their users: A survey of demographics and ideologies
Source: PLoS One. 2018 Jul 11;13(7):e0198033. doi: 10.1371/journal.pone.0198033 (PMC6040870; doi:10.1371/journal.pone.0198033)
Supplement: S29 Table — (PDF) [file pone.0198033.s030.pdf]

Table S29. Logistic regression on ideology — Online privacy, visibility, and scholarly identity.

	Maintaining my privacy online is very important to me. (n = 1112)		Having a profile on Mendeley/Zotero makes me more visible in my field. (n = 736)		Being visible online is critical for my scholarly identity. (n = 874)	
	Coef.	(S.E.)	Coef.	(S.E.)	Coef.	S.E.
(Intercept)	2.49***	(0.66)	-0.99**	(0.38)	1.27***	(0.37)
Platform						
Zotero	0.59	(0.35)	-1.48***	(0.20)	0.20	(0.18)
Education						
High School	-0.79	(1.09)	0.34	(0.67)	-0.18	(0.65)
Bachelor	-0.53	(0.51)	0.32	(0.30)	-0.23	(0.29)
Doctorate	-0.52	(0.40)	-0.33	(0.22)	0.19	(0.20)
X^2	2.4		4.5		1.9	
Discipline						
Arts & Humanities	0.87	(0.60)	0.19	(0.29)	-0.47	(0.26)
Computer Sciences	1.13	(1.06)	-0.40	(0.47)	-0.31	(0.41)
Engineering	1.22	(0.77)	-0.01	(0.33)	-0.52	(0.32)
Environmental Sciences	0.49	(0.79)	0.39	(0.47)	-0.76	(0.44)
Life Sciences	0.33	(0.41)	0.01	(0.25)	-0.40	(0.24)
Mathematical Sciences	-0.34	(1.12)	-0.48	(0.85)	-1.05	(0.62)
Physical Sciences	1.58	(1.05)	-0.08	(0.44)	-1.48***	(0.37)
Psychology	0.03	(0.67)	0.08	(0.42)	-0.42	(0.37)
None	-0.65	(0.91)	-14.84	(474.56)	-0.01	(0.64)
Others	-0.24	(0.51)	0.50	(0.30)	0.02	(0.31)
X^2	9.3		6.0		20.5*	
Occupation						
Professor	-0.17	(0.52)	0.16	(0.29)	0.20	(0.28)
Lecturer	15.23	(804.66)	1.31***	(0.39)	0.08	(0.36)
Researcher	-0.34	(0.45)	-0.16	(0.26)	-0.05	(0.24)
Practitioner	0.23	(0.58)	0.47	(0.29)	-0.10	(0.27)
None	-0.76	(0.62)	-0.31	(0.47)	-0.67	(0.37)
X^2	2.8		17.4**		5.6	
Gender						
Female	0.52	(0.33)	-0.19	(0.18)	-0.14	(0.17)
Other	-0.65	(0.80)	-0.33	(0.63)	0.86	(0.79)
X^2	3.6		1.3		2.1	
Age	0.01	(0.02)	0.03**	(0.01)	0.00	(0.01)

Note: * $p < 0.05$ ** $p < 0.01$ *** $p < 0.001$ (two-tailed tests)
